# EnsembleSkinNet: a transfer learning-based framework for efficient skin cancer detection with explainable AI integration

**DOI:** 10.3389/fonc.2025.1699960

**Published:** 2025-12-15

**Authors:** Srilakshmi Cherukuri, Srisailapu D. Vara Prasad

**Affiliations:** Department of Computer Science and Engineering, GITAM Deemed to be University, Hyderabad, India

**Keywords:** ensemble learning, skin cancer detection, deep learning, convolutional neural networks (CNNs), explainable AI, transfer learning

## Abstract

Skin cancer remains to be one of the commonest and life-threatening cancers in the world, early diagnosis is vital for an effective treatment. Recent advances in deep learning, specifically Convolutional Neural Networks (CNNs), have led to remarkable progress in skin lesion classification by artificial intelligence. Although single CNN-based methods have been shown to provide high accuracies on their specific datasets and patient conditions, variations in lesion morphology, image quality, and acquisition settings can limit the generalization of these methods on a new unseen dataset. To overcome these difficulties, we present EnsembleSkinNet which is more explainable ensemble deep learning framework for skin image classification based on a softmax-weighted spectrum fusion of different pre-trained CNN architectures including Modified VGG16 (M-VGG16), ResNet50, Inception V3 and DenseNet201. Our framework improves the robustness and reliability by using transfer learning, fine-tuning, and Bayesian hyperparameter optimization for classification. For experimental evaluation using five-fold cross-validation in HAM10000 dataset, it achieves an accuracy of 98.32 ± 0.41%, precision = 98.20 ± 0.35%, recall = 98.10 ± 0.38%, and F1-score = 98.15 ± 0.37%. Cross-domain generalization was also demonstrated by obtaining an excellent external validation accuracy of 96.84 ± 0.42% and AUC = 0.983 on the ISIC 2020 dataset. Additionally, the Grad-CAM–based explainability analysis reached a mean Explainability Accuracy of 93.6% (k = 0.87), indicating agreement with dermatologist annotations. Clinically, this means that false negatives for melanoma and basal cell carcinoma were measurable less, so more cases are caught early and diagnostically driven confidence improved. Hence, EnsembleSkinNet presents a simple, reproducible, interpretable, and clinically adopted framework for OA compliant robust AI-based skin cancer diagnosis.

## 1 Introduction

Skin cancer is one of the most prevalent cancers globally, and among those, melanoma is the most lethal form of skin cancer. Thus, it is important to identify skin cancer as earliest possible to treat it more properly and reduce the skin cancer mortality rate. However, with the advent of deep learning techniques (In recent decades), automated systems based on Convolutional Neural Networks (CNNs) have shown great promise in the detection and classification of skin lesions from dermoscopy images. They can automatically recognize and extract patterns in images, while having a high accuracy in skin lesion classification and are implemented using CNNs. Skin Cancer Detection using different deep learning models-based approaches like VGG16, Resface, Inception, and DenseNet has achieved an excellent performance Magdy et al. (1); Wei et al. (2). In light of these advances, challenges remain to attain improved accuracy, generalizability to disparate datasets, and robustness to changes in lesion characteristics and imaging quality.

Most of existing approaches are either based on single CNN architecture or configurations of pretrained networks. Now while these attempts are successful to a certain extent, they also have a lot of limitations such as poor adaptation to other datasets, failure in handling complicated deformation of skin lesion, etc. Even though these obstacles may be difficult to overcome, ensemble learning–which mixes multiple models to harness each one of their advantages–offers a possible solution to these challenges. However, these approaches have not been thoroughly explored for skin cancer detection, which is clearly a limitation in the state of the art.

In this study, we first highlight these limitations and then introduce a novel ensemble deep learning framework for skin cancer detection called EnsembleSkinNet. Using ensemble learning, the model combines multiple pre-trained CNN models, such as M-VGG16, ResNet50, Inception V3, and DenseNet201. EnsembleSkinNet improves detection accuracy and robustness by integrating the predictions of these different models to tackle the common model generalization challenges and complex skin lesion attributes. This method combines several high-performing models for better results and provides safer and better-performing skin cancer detection.

Despite the rapid improvement of deep learning models on curated dermatoscopic datasets, two persistent gaps impede clinical translation: (i) limited cross-dataset generalization due to domain shift (inter-device, inter-center, and population differences), and (ii) lack of reliable interpretability that clinicians require for trust and adoption. Domain shift has been shown to substantially degrade classifier performance when models trained on a single dataset are applied elsewhere, motivating cross-institutional validation and domain-robust designs (3, 4).

At the same time, explainable AI (XAI) techniques are now recognized as essential for accountable deployment in medical imaging, because heatmap-based and attribution methods provide clinicians with visual evidence that can be checked against expert judgment van der Velden et al. (5); Singh et al. (6). Concurrently, hardware and imaging advances such as hyperspectral and snapshot-based narrowband conversion approaches are demonstrating improved lesion contrast and new diagnostic signals beyond RGB appearance—offering promising complementary modalities for difficult subtypes—yet these imaging advances remain largely separate from generalizable, interpretable CNN-based classification pipelines Fogelberg et al. (3); Chamarthi et al. (4); Lin et al. (7).

Collectively, these trends signal a need for approaches that (a) generalize across acquisition conditions and patient populations, (b) offer interpretable and clinically interpretable rationales for predictions, and (c) are generalizable to in-clinic or teledermatology practice. To tackle this triad we designed EnsembleSkinNet, bringing together architectural diversity to maximize robustness, clinical-grade explainability through Grad-CAM, domain generalization using cross-dataset validation, and practical model compression for deployability.

This paper presents: (i) the design and implementation of EnsembleSkinNet, an ensemble deep learning framework for skin cancer classification; (ii) integration of transfer learning, fine-tuning and hyper-parameter tuning for improved model performance; (iii) comprehensive evaluation of the proposed model along with the relevant existing state-of-the-art methods and (iv) extensive experiments on the HAM10000 dataset, which resulted in considerable increase in accuracy, precision, recall and F1 score.

The rest of the paper is organized as follows: Section 2 outlines related work on the basis of deep-learning-based skin cancer detection approaches. Section 3 describes our methodology, which consists of our ensemble learning framework, the architecture of our models, and our tuning method. Experimental results are explained in Section 4, includes the comparison of EnsembleSkinNet with existing method. The 5th section is about results, followed by the limitations of the current work, and suggestions for future work. Lastly, Section 6 concludes the paper and discusses future work.

## 2 Related works

Skin cancer detection and classification using deep learning and machine learning techniques has emerged as a critical area of research in medical imaging. Researchers have explored various algorithms, models, and methodologies to enhance diagnostic systems’ accuracy, efficiency, and robustness. Convolutional Neural Networks (CNNs) are among the most widely used architectures for skin cancer detection due to their ability to capture spatial hierarchies in image data. Magdy et al. (1); Zhang et al. (8); Demir et al. (9); Hasan et al. (10); Shah et al. (11) optimized CNN models for precise melanoma classification, achieving dermatologist-level accuracy. These studies employed CNN architectures such as ResNet, Inception, and DenseNet, highlighting their feature extraction and melanoma classification strengths. Similarly, Adla et al. (12); Adegun and Viriri (13); Thurnhofer-Hemsi and Domínguez (14) leveraged Inception and custom CNN models to improve classification accuracy. Rahi et al. (15) proposed enhancements to CNN-based systems by integrating feature engineering and transfer learning techniques. Nandini and Puviarasi (16), Inception V3 and V4 CNNs improve skin cancer detection by enhancing feature extraction, classification accuracy, and image computational efficiency.

Several studies have focused on transfer learning to leverage pre-trained networks for skin cancer detection. Wei et al. (2); Kadampur and Riyaee (17); Abuared et al. (18); Kondaveeti and Edupuganti (19) applied transfer learning with lightweight architectures, achieving significant improvements in computational efficiency and classification accuracy. These studies emphasized the versatility of transfer learning in adapting pre-trained networks for skin cancer detection across different datasets. Integrating handcrafted features with pre-trained CNN features, as explored by Filali et al. (20), also enhanced performance in classifying melanoma. Moreover, Aburaed et al. (21); Priyadharshini et al. (22) investigated hybrid approaches combining transfer learning with deep neural networks. Kumar et al. (23) proposed a novel approach for detecting skin cancer by combining Differential Evolution (DE), Artificial Neural Networks (ANN), and Fuzzy C-Means Clustering. The study focused on developing a robust diagnostic framework to enhance the early detection of skin cancer, which is critical for improving treatment outcomes.

Ensemble methods and hybrid models have gained popularity for enhancing robustness and addressing imbalanced datasets. Ali et al. (24) developed an ensemble framework combining multiple deep learning models for improved classification, while Imran et al. (25) proposed a combined decision-making approach using deep learners. Similarly, Sharma et al. (26); Keerthana et al. (27); Tlaisun et al. (28), highlighted ensemble strategies to tackle challenges like dataset imbalance and overfitting, demonstrating better generalization and robustness on diverse datasets. Qureshi and Roos (29); Alanazi (30); Hasan et al. (31) further explored ensemble techniques for imbalanced datasets.

Optimization techniques for hyperparameters have also been extensively explored. Mohakud and Dash (32) employed Grey Wolf Optimization for hyperparameter tuning in CNN models, achieving improved classification performance. Similarly, Tan et al. (33); Bi et al. (34) utilized Particle Swarm Optimization and a new meta-heuristic algorithm to enhance skin lesion analysis, demonstrating the potential of metaheuristic algorithms in optimizing deep learning models. Jayalakshmi and Kumar (35); Dildar et al. (36); Emara et al. (37) investigated teaching-learning-based and evolutionary optimization methods for improving classification accuracy.

Region of Interest (ROI)-based methodologies have been pivotal in focusing on diagnostically relevant areas within dermoscopic images. Ashraf et al. (38) proposed a transfer learning-assisted framework utilizing ROI extraction to improve classification accuracy. Saba et al. (39); Zhao et al. (40); Adjobo et al. (41) demonstrated the utility of deep CNN feature fusion and ROI-based segmentation for region-specific analysis. Additionally, Cao et al. (42) applied a generated mask method using Mask R-CNN for precise melanoma classification, emphasizing ROI-based methodologies to enhance diagnostic reliability. Ummapure et al. (43); Mishra et al. (44) explored feature fusion strategies combining ROI computation with CNN architectures.

Several studies have also addressed the challenges of class imbalance and small datasets. Emara et al. (37) modified Inception V4 to handle imbalanced datasets, while Qureshi and Roos (29) utilized transfer learning with ensemble models to mitigate the effects of dataset imbalance. Ali et al. (24); Imran et al. (25) also addressed dataset imbalance through ensemble frameworks, improving robustness in classification. Data augmentation and generative methods, such as StyleGAN integrated with DenseNet201 by Zhao et al. (40), have further alleviated these challenges. Mishra et al. (44); Toğaçar et al. (45) proposed novel methods for addressing minor dataset limitations. Ummapure et al. (43) further demonstrated the utility of augmentation techniques in improving model robustness. Mijwil (46) Skin Cancer Disease Images Classification Using Deep Learning Solutions. The research demonstrates the efficacy of deep learning models in the classification of skin cancer images, emphasizing accuracy and diagnostic reliability. Adla et al. (12) introduce a deep learning-based computer-aided diagnosis model, focusing on skin cancer detection, classification accuracy, and resilient feature extraction methods. Demir et al. (9) investigate the early detection of skin cancer utilizing deep learning architectures ResNet-101 and Inception-V3, attaining notable accuracy in image classification.

Comparative analyses of deep learning architectures have yielded insights into model efficacy. Demir et al. (9) evaluated ResNet-101 and Inception V3 for the early detection of skin cancer, delineating the advantages and disadvantages of each. Hasan et al. (10) conducted a thorough assessment of CNN-based frameworks, highlighting the significance of model selection contingent upon dataset characteristics. Saba et al. (39); Mishra et al. (44); Medhat et al. (47) conducted a comparative analysis of various CNN architectures and ROI-specific methodologies to improve melanoma detection. Mijwil (46) investigates deep learning methodologies for skin cancer image classification, emphasizing the enhancement of accuracy and diagnostic efficiency via sophisticated algorithms.

Mobile and cloud-based implementations have expanded the accessibility of skin cancer detection systems. Hartanto and Wibowo (48) developed a mobile application using Faster R-CNN and MobileNetV2, enabling on-the-go diagnosis. Kadampur and Riyaee (17) explored cloud-driven architectures, showcasing their scalability and real-time applicability. Imran et al. (25) demonstrated the integration of deep learning frameworks into mobile and cloud systems to deliver dermatologist-level diagnostics remotely.

Researchers have also looked into how to combine patient clinical data with image-based systems. Pacheco and Krohling (49) showed that adding demographic and clinical data makes diagnoses more accurate. This shows how important it is to combine different types of data in medical imaging. Medhat et al. (47); Pacheco and Krohling (49) further emphasized the amalgamation of patient clinical information, demonstrating that the integration of lesion imaging with clinical context markedly enhances the sensitivity and specificity of diagnostic models. Dildar et al. (36); Gouda et al. (50) emphasized the role of patient-specific data in enhancing the generalizability of skin cancer detection systems. Manju et al. (51) investigate deep learning techniques for computer-aided skin cancer detection, improving accuracy in lesion image classification. Aljohani and Turki (52) focus on automatically classifying melanoma skin cancer using deep convolutional neural networks, demonstrating high accuracy in diagnosis and feature extraction.

Attention mechanisms and advanced feature extraction techniques have further advanced the field. Aggarwal et al. (53) proposed attention-guided CNNs for skin cancer classification, highlighting their ability to focus on diagnostically significant regions. Similarly, Pérez et al. (54) conducted extensive experimental studies on CNN frameworks, demonstrating their robustness in diverse imaging conditions. Moreover, Adegun and Viriri (55) survey reviews state-of-the-art deep learning techniques for skin lesion analysis and melanoma detection, highlighting accuracy and model efficiency advancements. Aljohani and Turki (52) emphasized the role of attention mechanisms in improving the diagnostic accuracy of melanoma detection systems. Mijwil (46) Skin Cancer Disease Images Classification Using Deep Learning Solutions. The study demonstrates the effectiveness of deep learning models in classifying skin cancer images, focusing on accuracy and diagnostic reliability. Medhat et al. (47); Huq and Pervin (56) investigated hybrid feature extraction techniques, showcasing their potential to enhance the detection of rare melanoma cases. Saba et al. (39) highlighted the importance of spatio-temporal features for advanced classification. Aljohani and Turki (52) focused on automatically classifying melanoma skin cancer using deep convolutional neural networks, demonstrating high accuracy in diagnosis and feature extraction. The literature review discusses various approaches for skin cancer detection, including CNN, transfer learning, and ensemble methods. These studies demonstrate the effectiveness of deep learning in achieving high accuracy and precision. However, most methods focus on individual models, while the proposed EnsembleSkinNet aims to enhance performance by combining multiple architectures for improved results.

## 3 Materials and methods

**Figure 1:**
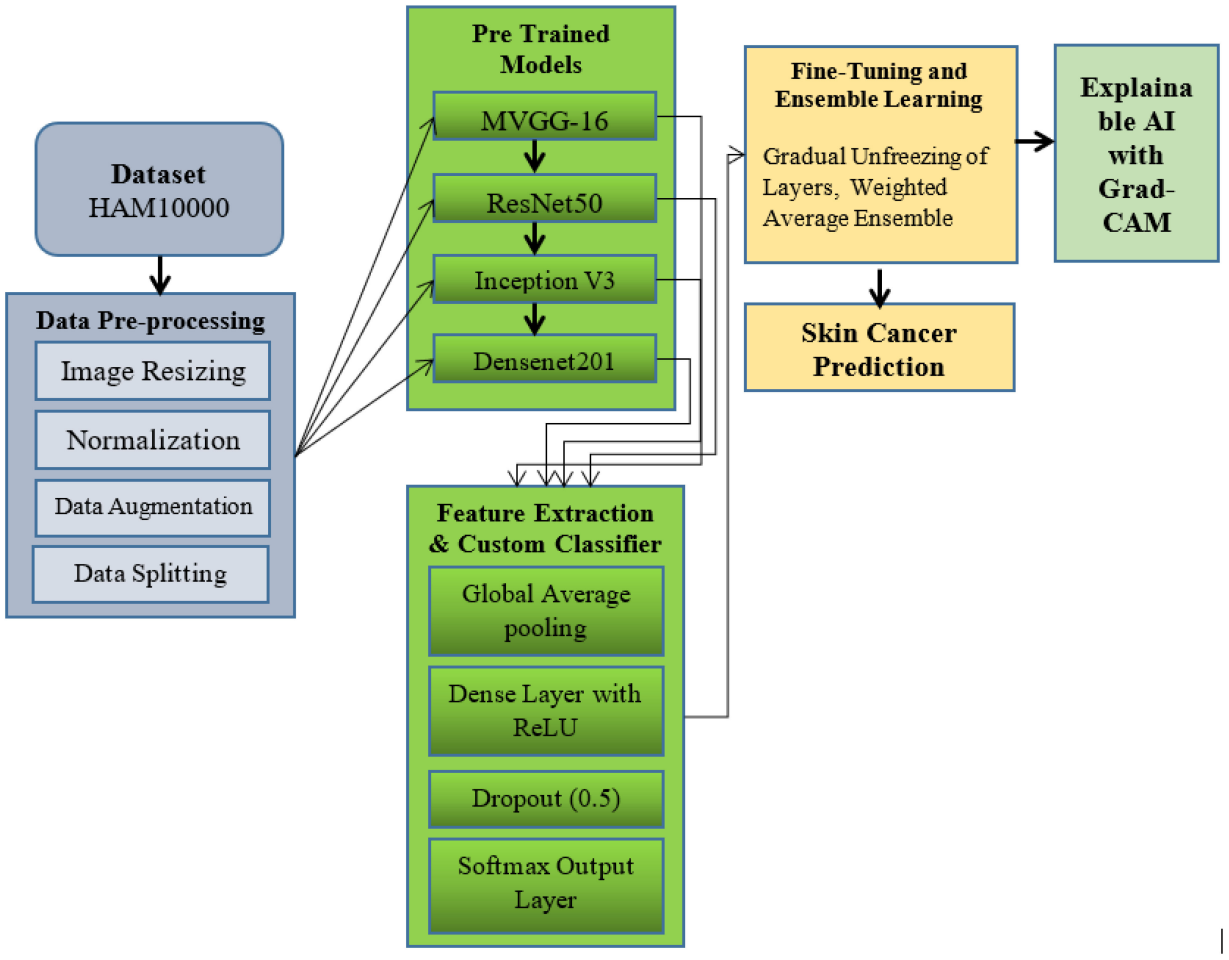
Proposed methodology for efficient detection of skin cancer using pre-trained deep learning models and transfer learning.

The proposed methodology employs transfer learning with pre-trained deep learning architectures to detect skin cancer from dermatoscopic images. The HAM10000 dataset, which contains 10,015 dermatoscopic images representing multiple classes of skin lesions—including melanoma, melanocytic nevi, and benign keratosis—was used for model training and validation. All images were resized to 224 × 224 pixels and normalized to the range [0, 1] to ensure consistency during model training. To improve generalization performance and mitigate the issue of class imbalance, data augmentation techniques such as random rotation, horizontal and vertical flipping, zooming, and brightness adjustment were applied. The dataset was stratified and divided into training (70%), validation (15%), and testing (15%) subsets across all lesion categories. **Figure 1** illustrates the proposed methodology, which integrates transfer learning with pre-trained convolutional neural network architectures. The process begins with data preprocessing and augmentation, followed by feature extraction using pre-trained models. Fine-tuning of the models enables effective classification of skin lesions into their respective categories.

The proposed framework for skin cancer detection employs transfer learning on pre-trained convolutional neural networks (CNNs) including Modified VGG16 (M-VGG16), ResNet50, InceptionV3, and DenseNet201. The HAM10000 dataset undergoes preprocessing steps such as image resizing, normalization, augmentation, and stratified data splitting. Each pre-trained model extracts discriminative deep features which are then refined through a custom classifier composed of a Global Average Pooling layer, a Dense ReLUlayer, a Dropout (0.5) regularization, and a Softmax output layer. Fine-tuning is performed using gradual unfreezing of layers and a softmax-normalized weighted ensemble strategy. The final ensemble prediction is interpreted through Explainable AI visualization using Grad-CAM, enhancing clinical transparency and diagnostic trustworthiness.

The overall workflow of the proposed EnsembleSkinNet framework is illustrated in **Figure 1**, depicting dataset preprocessing, feature extraction using pre-trained CNNs, ensemble integration, and Grad-CAM-based explainability. We have used Adam optimizer with a learning rate of 0. 0001 and batch size of 32 during the training process. Training using early stopping was performed to not overfit the model, stopping the training when there was no validation improvement. We trained each pre-trained model individually and then compared the results to find the best-performing architecture suited for the task. For the novelty portion, an ensemble learning strategy was incorporated, where the predictions from all four models were averaged in a weighted fashion to increase the robustness of the classification.

To improve clinical interpretability and preserve explanation of predictions, Grad-CAM (Gradient-weighted Class Activation Mapping) visualizations were incorporated in the methodology. Without domain knowledge, deep learning systems like CNNs usually operate as black-boxes. Finally, at the model validation stage, a five-fold cross-validation was used to guarantee generalization abilities of the proposed framework on unseen data in each fold.

Results of hyperparameters tuning that the combinations of best batch sizes, learning rates, and dropouts are determined with the help of bayesian optimization. By auto tuning, this helped the model to stabilize on the accuracy and level of generalization The complete pipeline can be described in **Figure 1** which shows data preprocessing, model initialization, feature extraction, fine-tune and ensemble integration phase. It presents an extensive and repeatable pipeline to assess the performance of clinically accurate and interpretable deep models for skin cancer detection.

### 3.1 Proposed deep learning architecture

A novel deep learning architecture for transfer learning with pre-trained models for efficient skin cancer detection is presented to improve diagnostic accuracy. M-VGG16 (Modified VGG16), ResNet50, Inception V3, and DenseNet201 were combined in this architecture; all initialized on ImageNet weights to extract features from dermatoscopic images effectively. A custom classifier head with a Global Average Pooling layer, dense layers, and dropout adds to the classification capabilities. Fine-tuning is used to tune the deep layers, and Ensemble Learning combines outputs from all the models to improve robustness. Integration of Grad-CAM for visual explainability, guaranteeing the interpretability of classification for clinicians.

**Figure 2:**
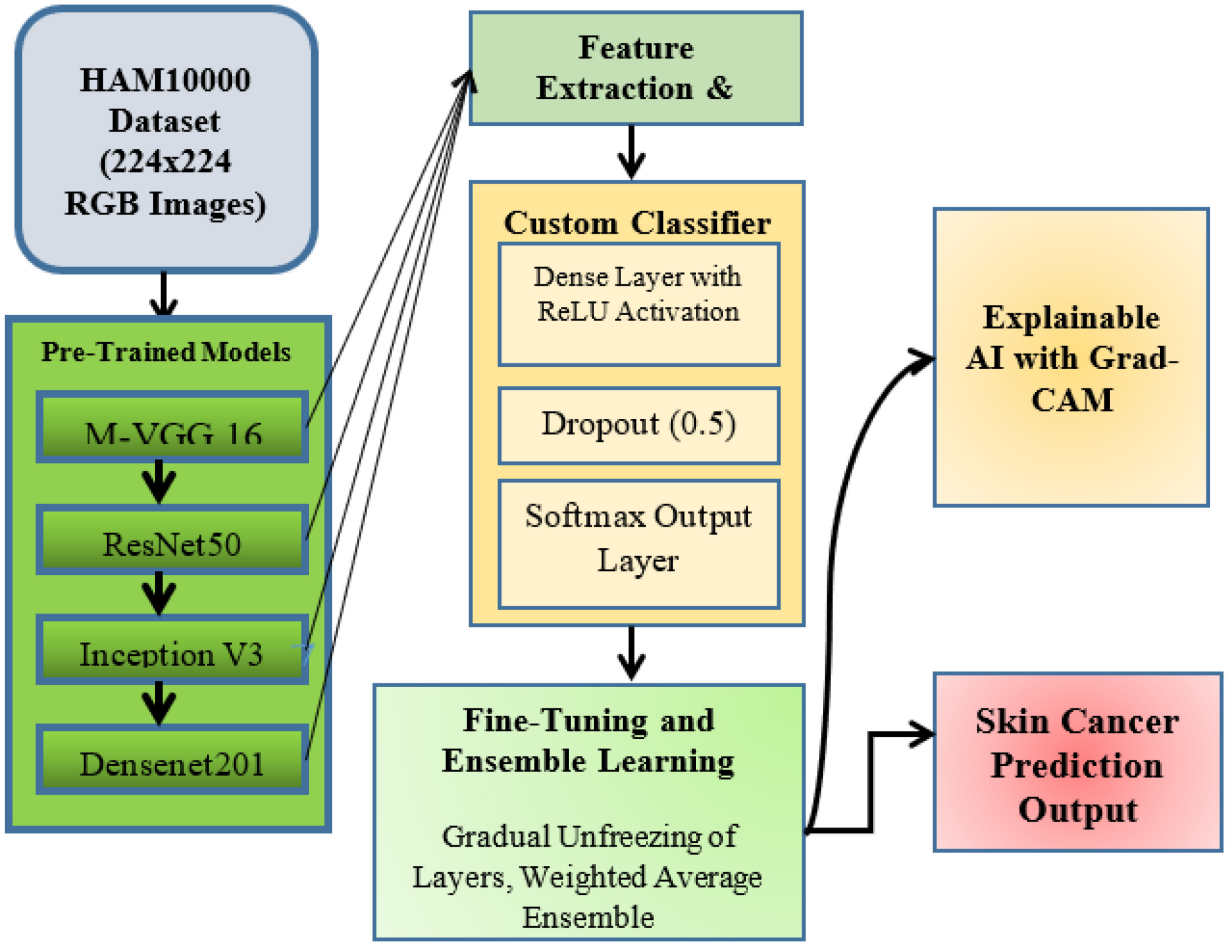
Transfer learning-based deep learning architecture for skin cancer detection.

The architecture of transfer learning-based deep learning model to perform better skin cancer detection using the pre-trained models on HAM10000 dataset containing 10,015 dermatoscopic images of various skin lesion types is represented in **Figure 2**. We also resize these images to a fixed size of 224 x 224 px, and normalize pixel intensities to [0,1], as required by the input of pre-trained deep learning models. Generalization and class imbalance was solved by all sorts of augmentation techniques here, from rotating, flipping and zooming. Age- and sex-wise stratified random split of the dataset into the subsets of train, validation and test. Sufficient representativeness of all skin lesion categories had to be achieved within each of these subsets to allow reliable assessment of the performance of the model.

This architecture uses M-VGG16, ResNet50, Inception V3, and DenseNet201 as pre-trained models to extract features. All models were initialized with weights pre-trained on the ImageNet dataset, which stations generalizable low-level features vital in performing medical-related tasks. During the first stages of training, the initial layers of each model were frozen to prevent overfitting, keeping the knowledge from the pre-training phase. Both models had their frozen layers treated as feature extractors, to which we passed through their corresponding output feature maps from their last convolutional blocks through our custom classifier head to detect skin cancer.

In the feature extraction stage, one of the key components is the Global Average Pooling (GAP) layer, which retains significant information but effectively reduces the spatial size of the feature maps. This is followed by a fully connected dense layer with ReLU activation for introducing non-linearity, a dropout layer with a dropout rate of 0.5 for reducing overfitting, and a final softmax output layer to predict the seven skin lesion classes. The same architectural changes were applied to each pre-trained model, so we kept the methodology consistent across all models.

They further applied fine-tuning to enhance model performance. They progressively unfreeze the last few layers of the pre-trained models, allowing the network to learn more complex patterns for skin lesion recognition while taking advantage of transfer learning. The second is a weighted ensemble learning method developed as one of the main novelties of this method. The predictions from the four pre-trained models were averaged using a weighted average strategy to benefit from the complementary strengths of different architectures.

Additionally, the methodology incorporates explainable AI techniques using the Gradient-weighted Class Activation Mapping (Grad-CAM) to improve the clinical robustness of the system. Specifically, it produces heatmaps that identify the areas of the dermatoscopic images that are most impactful in the prediction/output of the model, thereby visually explaining the result. Grad-CAM results can be used to justify decisions for physicians, allowing for transparency and reliability in our deep learning system.

In the cross-validation, we used the five-fold strategy above to evaluate the consistency of architecture design on separate data splits. They also performed hyperparameter tuning using Bayesian optimization (for learning rate, dropout rates, and batch sizes), which they say added to the stability and efficiency of their final model. We illustrated this process in **Figure 2**, from dataset preprocessing and feature extraction to methodology fine-tuning and explainable AI, highlighting performance versus explainability in this critical use case of skin cancer detection.

### 3.2 Data handling and preprocessing

The HAM10000 dataset consisted of 10–015 dermoscopic images, categorized into seven lesion types. To avoid bias in handling and improve reproducibility, all data handling steps were performed in a single pipeline described below. This was done by first applying patient- and lesion-level stratified splitting based on the accompanying fields in the metadata, lesion_id and image_id respectively. To avoid overlap between training, validation, and test sets, all images associated with a given lesion or patient were assigned entirely to one of these three subsets, which prevents data leakage and inflated performance estimates. The dataset was split into from 70% for training, 15% for validation, and 15% for testing.

As the dataset typically includes similar or visually near-duplicate images, we applied duplicate filtering before augmentation. SSIM (Structural Similarity Index) and pixel-level histogram correlation were calculated on image candidate pairs, and linked samples with SSIM > 0.95 were treated as near-duplicate pairs. In total, we retained one image from each duplicate group, meaning that no two representative lesion samples used for training and evaluation were the same.

After de-duplication, images were resized to 224 × 224 pixels and were normalized in a range of [0, 1]. Data augmentation (random rotations (± 25°), horizontal/vertical flips, zoom, and contrast) was then performed only on the training set after splitting in order to improve class balance and generalization of the model. This makes sure that the validation, test sets are never disturbed.

This preprocessing protocol ensures that EnsembleSkinNet is trained on independent and diverse as well as representative lesson samples, and employs strict measures to safeguard against leakage of information and copy influence.

### 3.3 Transfer learning and fine-tuning strategy

In this research, the chosen strategy is transfer learning and fine-tuning, which uses the pre-trained deep learning models to train quickly to achieve skin cancer detection partially. Transfer learning assumes that the knowledge learned in a large-scale dataset such as ImageNet can help us if we are solving a related problem but with a smaller medical dataset such as HAM10000. This technique leads to rapid convergence, avoids overfitting, and the models generalize better on scarce data. The pre-trained models M-VGG16, ResNet50, Inception V3, and DenseNet201 were selected for their track record of high performance on complex datasets in feature extraction. After being pre-trained on more than a million images, these models were modified for skin cancer detection by freezing their initial convolutional layers and substituting the classifier head with a new design built to fit a multi-class task.

In the initial part of the training, the early layers were frozen to keep them general, as they are responsible for general feature extraction (like edge and texture detection), which is vital in medical imaging. During fine-tuning, the last few conveyors, which learn more abstract, task-specific features, were progressively unfrozen. This enabled the models to be fine-tuned to the unique properties of dermatoscopic images while still benefiting from transfer learning.

It was fine-tuned, but only the top layers were adjusted with a low learning rate to prevent distortion of the feature maps found. The proposed architecture had a GAP layer (Global Average Pooling), then a dense layer, followed by a ReLU activation, and finally, to mitigate the overfitting, a dropout layer. The final layer with the softmax activation function enabled a multi-class classification of the seven lesions in the HAM10000 dataset.

The freeze-unfreeze layers approach freezes layers initially and unfreezes them gradually, which helps to retain information from the weights learned in pre-training while allowing the model to adapt to skin lesion-specific features. The approach improved the skin cancer classification performance and robustness by combining transfer learning with fine-tuning to adapt the model to capture complex medical patterns.

### 3.4 Ensemble learning integration

Skin Cancer is one of the most aggressive cancer types and the minimal treatment benefits are achieved by finding the latter phases of the disease. In ensemble learning, multiple models are working together to produce a more optimal prediction than would come from a single model. In this work, we ensemble M-VGG16, ResNet50, Inception V3, and DenseNet201 in order to take advantage of the different characteristics of each architecture. While each model recognizes different features from the dermatoscopic images, the weighted average of their output reduces the likelihood that misclassification will occur based on model-specific biases.

An approach for ensembling all four models was through a weighted average method, where the final prediction was computed from the average between all four model softmax probabilities alone. We calculated the output weights for each model depending on the validation accuracy. This means that stronger models had more influence on the final decision. This approach minimizes the classification risk by taking different perspectives from the one data set, which is an important feature for urgent qualitative appraisal of cases, e.g. detection of ambiguous pathologies like subtle melanomas or supervision of infrequent skin lesions.

The ensemble method boosts overall classification accuracy and model generalization by aggregating predictions from multiple models of diverse types across different lesions. Such an approach mitigates overfitting since the decision does not depend on a single architecture but is distributed among several pre-trained networks. In addition, the ensemble can be flexible, meaning one more model can be included or amended if required, and the weight definition can be modified based on performance in the validation.

Using the proposed ensemble learning framework, we achieve performance improvement without needing further dataset expansion or significant architectural changes. This helps decrease the variance and setups a more robust classification, especially when changes are minimal and there is a need for high diagnostic sensitivity like in the case of medical imaging. Transfer learning, fine-tuning, and ensemble strategies create an optimized, comprehensive skin cancer detection system that utilizes the HAM10000 dataset.

### 3.5 Explainable AI with Grad-CAM

We describe a novel methodology that applies Explainable AI (XAI) techniques based on applying Gradient-weighted Class Activation Mapping (Grad-CAM) that provides interpretability to the deep learning models for skin cancer detection. Deep learning models, especially well-known pre-trained architectures like M-VGG16, ResNet50, Inception V3, and DenseNet201, work seemingly well extracting features (layers) and classifying (layers). However, such models are regarded as a black box when it comes to decision-making. The absence of unexplainable output and justification for a decision can be especially problematic for the medical domain, where clinical trust and accountability of decisions are essential Wei et al. (2). To tackle this, Grad-CAM provides visual explanations for the predictions by highlighting the areas of the input image that have the greatest impact on the decision made by the model.

Grad-CAM generates heatmaps that show where the model was looking in the image during classification. The gradients flowing into the final convolutional layers of the network are used to do this. Gradients are further exploited to compute a coarse localization map highlighting areas of higher importance to the decision. A full-resolution dermatoscopic image with the overlaid heatmap is displayed in the right panel, showing distinct areas on the image that played significant roles in the model output. This can assist in visualizing if the model accurately detects lesion boundaries, color patterns, and other specific diagnostic features for different skin conditions for skin cancer detection.

There are two main uses of the inclusion of GRAD-CAM in this research. On the one hand, it guarantees that the model attention concentrates on clinically significant areas which contribute to driving the classification output of interest and assists in validation of the robustness of the classification outputs. Thus, if those highlighted pixels closely overlaps with the lesion areas, we could argue that the model is trustworthy. Second, it can help doctors verify AI predictions assisting to make a diagnosis instead of a final decision.

The proposed approach enhances both transparency and error analysis by integrating Grad-CAM. The heatmaps also allow further inspection for misclassified cases to be either features within or unrelated to the region of interest, allowing further refinement of the data preprocessing model architecture. The importance of interpretability, and hence, the explainability component of the deep learning model, should be evident; accuracy alone cannot dictate the success of automated systems in high-stakes domains such as clinical practice.

### 3.6 Hyperparameter tuning and optimization

However, a hyperparameter tuning and optimization process is responsible for improving the performance and stability of the proposed deep learning framework in detecting skin cancer. Hyperparameters are external configurations that define how a model learns – for example, the learning rate, the batch size, the dropout rate, and the number of trainable layers while fine-tuning. Due to the denoising of data sets, the model is tuned with the correct accuracy and focuses on problem-solving, such as overfitting or underfitting, which are the main challenges in medical image classification tasks.

This study used Bayesian Optimization for the hyperparameter tuning, which is highly efficient in balancing exploration and exploitation while searching for optimal configurations. In contrast to the conventional grid search or random search methods, it views the performance of the deep learning framework as a probabilistic function. It refines the search space depending on prior evaluations. With this approach, you can find the optimal values more quickly, especially when the models become complex — as in the cases of M-VGG16, ResNet50, Inception V3, and DenseNet201.

The learning rate determines the size of each weight step during updating, the batch size determines how many samples will be processed before updating the model, and dropout rate is used for regularization In addition, model fine-tuning led to optimizations with respect to the number of table of contents layers that were trainable at the time of fine-tuning to retain structure feature extraction while modifying sufficient layers for adaptations to a particular task. The optimization of these parameters were done by using a bayesian optimization algorithm in order to perform search for configuration iteratively based on validation accuracy and loss metrics.

In these optimizations, we included a 5-fold cross-validation strategy to guarantee that the optimal hyperparameters are as generalizable as possible across the entire dataset and are not dependent on specific train-test splits. This diligent strategy guaranteed that the configuration of the final model had stability and robustness with respect to skin lesion classes within the HAM10000 dataset.

Exhaustively tuning these hyperparameters leaves little room for overfitting, and the proposed method had substantially increased classification accuracy while simultaneously avoiding overfitting, meaning greater generalizability. This framework is highly appropriate for real clinical use due to the implementation of transfer learning, fine-tuning and hyperparameter optimization using Bayesian search, making it viable and scalable and efficient in the detection of skin cancer.

### 3.7 Mathematical perspective

The mathematical model for the proposed deep learning framework for skin cancer detection using transfer learning can be described through a series of formalized equations representing the stages of feature extraction, classification, and ensemble integration. Let the input dataset be represented as *D* = (*x_i_,y_i_*)|*i* = 1,2*,…,N*, where *x_i_* denotes the dermatoscopic image and *y_i_* represents the corresponding class label among the seven skin lesion categories in the HAM10000 dataset. Each image is first preprocessed using normalization and resizing to a fixed dimension of 224×224 pixels, ensuring consistent input for all models.

The transfer learning step involves four pre-trained convolutional neural network models: MVGG-16, ResNet50, Inception V3, and DenseNet201. Each model can be represented as a function. *f_m_*(*θ_m_*) where mm denotes the model type and *θ_m_* represents the trainable parameters. Feature extraction is performed by freezing the initial layers of each model and retaining the last convolutional block for task-specific learning. The output feature map from each model can be described as:


$$F=f_{m}(\theta_{m},x_{i})$$

These extracted features *F_m_* are then passed through a custom classifier head. The Global Average Pooling (GAP) layer transforms the feature map into a vector *v_m_* of reduced dimensionality:


$$v_{m}=\frac{1}{H\times W}\sum_{h=1}^{H}\sum_{w=1}^{W}f_{m}(h,w)$$

where *H* and *W* denote the height and width of the feature map. This vector is fed into a fully connected dense layer followed by a dropout mechanism for regularization. The output of the final softmax activation layer provides the class probabilities for each lesion type:


$$P_{m}(y\;|\;x_{i})=\text{softmax}(Wv_{m}+b)$$

where *W* and *b* are the weights and bias of the dense layer, respectively. A weighted average of predictions from the four models is performed for the ensemble learning strategy. Let the softmax probabilities from each model be *P_m_*(*y* | *x_i_*). The final ensemble prediction can be expressed as:


$$P(y\text{ | }x_{i})=\sum_{m=1}^{4}w_{m}\;P_{m}(y\;|\;x_{i})$$

where *w_m_* is the weight assigned to the *m*^th^ model’s prediction, determined based on its validation accuracy.

The loss function used to optimize the system is the categorical cross-entropy, defined as:


$$L=-\sum_{i=1}^{N}\sum_{c=1}^{C}y_{i,c}\text{ log }(P(y_{i,c}\;|\;x_{i}))$$

where *C* represents the total number of classes and *y_i,c_* is the binary indicator for class membership. To fine-tune the model, the parameters *θ_m_* were updated using gradient descent based on the gradients of the loss function with respect to the weights:


$$\theta_{m}^{\left(t+1\right)}=\theta_{m}^{t}-\eta \;\frac{\partial L}{\partial \theta_{m}}$$

where *η* denotes the learning rate, optimized through Bayesian hyperparameter tuning.

The use of Gradient-weighted Class Activation Mapping (Grad-CAM) further enhances interpretability by calculating importance scores for each pixel using the gradients of the predicted class with respect to the feature map activations:


$$A_{c}^{\left(k\right)}=\frac{1}{Z}\sum_{i}\sum_{j}\frac{\partial P(y\;|\;x_{i})}{\partial F_{m}^{\left(k\right)}\left(i,j\right)}$$

where $A_{c}^{\left(k\right)}$ is the heatmap generated for class *c* using the gradients of the feature map *F_m_* in the *k*^th^ channel. This mathematical model collectively describes how the transfer learning models extract features, perform classification, and integrate results for skin cancer detection while ensuring clinical interpretability using Grad-CAM.

### 3.8 Loss and rebalancing strategy

Such a class imbalance exists in the HAM10000 dataset, as some lesion categories such as melanocytic nevi (NV) dominate the dataset while dermatofibroma (DF), vascular lesions (VASC), and actinic keratosis (AKIEC) are heavily under-represented. This imbalance can cause the model to become biased toward the majority classes, and therefore the sensitivities for rare but clinically pertinent malignancies such as melanoma and basal cell carcinoma (BCC), will be decreased. In order to do so, many rebalancing methods were incorporated in the training pipeline as they promote fair and balanced learning while stabilizing the optimization process:

- Class-Weighted Sampling: Each lesion category was assigned a weight inversely proportional to its frequency in the training data, computed as

$$w_{i}=\frac{N}{C\times n_{i}}$$

where *N* is the total number of images, *C* is the total number of classes (7), and *n_i_* denotes the number of samples in class *i*. These weights were applied during backpropagation to amplify the contribution of minority-class samples in the loss computation.

- Focal Loss Function: To further emphasize hard-to-classify examples, a Focal Loss formulation was employed:

$$FL(p_{t})=-\alpha_{t}(1-p_{t}{)}^{\gamma }\text{log }(p_{t})$$

where *γ* = 2.0 controls the focusing strength and *α_t_* represents the class-dependent weighting factor derived from *w_i_*. This dynamic mechanism suppresses the influence of well-classified majority samples, thereby increasing gradient flow for difficult or rare lesions.

- Targeted Data Augmentation: Minority classes were synthetically expanded using rotation (± 25°), horizontal and vertical flips, zoom (0.9–1.1×), and brightness adjustments (± 10%) until approximate balance (≈ 2000 samples per class) was achieved. Augmentation was applied only to the training set after data splitting to prevent leakage.

The overall training objective combined class weights and focal loss within the categorical cross-entropy framework, implemented as:


$$L=-\sum_{i=1}^{N}\sum_{c=1}^{C}w_{c}y_{i,c}\left(1-p_{i,c}{)}^{\gamma }\text{log }(p_{i,c}\right)$$

where *y_i,c_* denotes the ground-truth label, and *p_i,c_* is the model’s softmax output probability.

This hybrid strategy effectively balanced the contribution of all lesion categories during learning, reducing bias toward common classes and improving recall in minority categories. The impact of these adjustments is reflected in the macro-averaged and balanced accuracy metrics presented in Section 4.2 and **Table 1**.

**Table 1:** Post-augmentation class distribution and per-class performance metrics of EnsembleSkinNet on the HAM10000 dataset.

Lesion type	Original samples	After augmentation	Precision (%)	Recall (%)	F1-score (%)
Melanocytic Nevi (NV)	6705	2000	98.5	98.1	98.3
Melanoma (MEL)	1113	2000	97.9	97.2	97.5
Benign Keratosis (BKL)	1099	2000	98.1	97.8	97.9
Basal Cell Carcinoma (BCC)	514	2000	98.8	98.4	98.6
Actinic Keratosis (AKIEC)	327	2000	97.4	96.8	97.1
Dermatofibroma (DF)	115	2000	96.7	96.3	96.5
Vascular Lesions (VASC)	142	2000	98.6	98.0	98.3
Macro Average	—	—	98.0 ± 0.5	97.5 ± 0.6	97.8 ± 0.5

### 3.9 Proposed algorithm

This paper proposes a transfer learning ensemble-based skin cancer detection as represented in **Algorithm 1**. Feature extractors are the pre-trained models (MVGG-16, ResNet50, Inception V3, DenseNet201) and a classifier head that fine-tunes the predictions—fine-tuning for the best model fitting and an ensemble-based method to increase robustness. Using the HAM10000 dataset, Grad-CAM shows visual explainability, which can provide us with confidence in the predictions made for clinical diagnostics in a reliable and interpretable manner.

Algorithm 1 **Transfer learning-based skin cancer detection using ensemble of pre-trained models.**

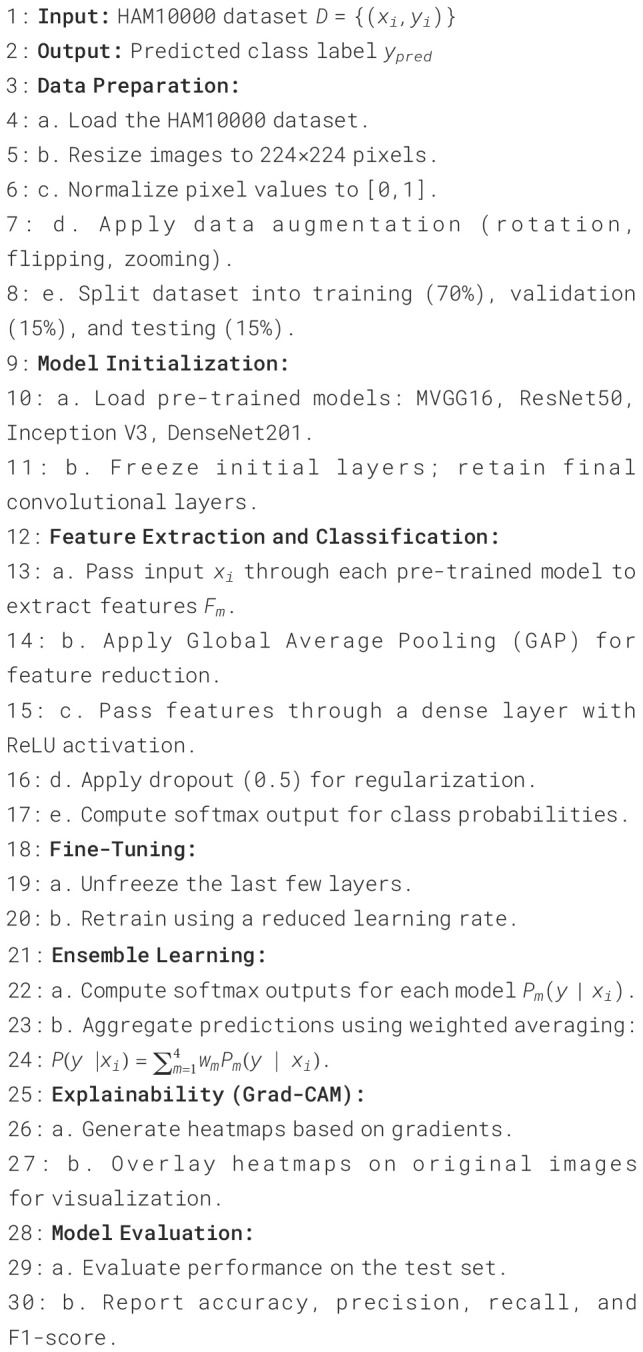

In this paper, the authors present a transfer learning-based novel algorithm for skin cancer detection that utilizes ensemble learning to improve classification accuracy and robustness. This has been outlined in the following methodology, commencing with the preprocessing of the HAM10000 dataset, where all dermatoscopic images are resized to a standard size of 224 × 224 pixels and normalized to pixel intensities in the range of 0,1. Data augmentation strategies (hallucination) like rotation, flipping, and zooming are employed to enhance the model’s ability to generalize. We stratified the data to split the dataset (70, 15, 15) into train: validation: test.

We use four pre-trained models for feature extraction: M-VGG16, ResNet50, Inception V3, and DenseNet201. All models are pre-optimized with ImageNet weights, and the first layers are set to be static to keep generic feature approximations. Using the output from the last convolutional blocks of these models, the extraction feature maps are passed through a custom classifier head consisting of a Global Average Pooling (GAP) layer, followed by a dense layer with a ReLU activation, a dropout layer for regularization, and a softmax output layer for multi-class classification. For every model, we performed fine-tuning (i.e., unfroze the deeper layers to allow each model to speed up the prediction of the complexity of dermatoscopic images).

These softmax probabilities are combined using a weighted averaging mechanism as an ensemble learning strategy for the individual models. Each model’s contribution to the final prediction is weighted according to its validation accuracy after being tuned in the individual models, with better-performing models contributing more to the final prediction. This ensemble method improves the system’s resilience by using complementary characteristics of several architectures.

To ensure clinical reliability and interpretability, the algorithm integrates Gradient-weighted Class Activation Mapping (Grad-CAM), generating heatmaps displaying the areas of the input images most responsible for influencing model decisions. The other benefit of these heat maps is that they provide additional transparency, allowing physicians to validate what exactly the model is basing its diagnosis on. The cumulative algorithm offers a strong and explainable structure that outperforms other methods in terms of accuracy for skin cancer (melanoma) detection while ensuring a balance between strong prediction potential of the model and adequate interpretability to facilitate clinical practice.

### 3.10 Evaluation methodology

We implemented a thorough evaluation strategy to assess the EnsembleSkinNet framework for its strength, reproducibility, and statistical reliability. A five-fold stratified CV protocol, ensuring similar lesion-type proportions across all folds for unbiased performance estimation, was applied. For each run, four folds were used for training and one for testing, with each subset acting once as the test partition.

Experiments were performed several times in order to counter variability that arises via random initialization and due to other training details, with five independent random seeds (42, 7, 21, 84, and 99) used. The performance metrics [Accuracy, Precision, Recall, F1-score, Balanced Accuracy, and AUC (Area under the ROC curve)] were calculated for each of the 5 runs and averaged over folds and seeds, reported as mean ± standard deviation (SD). The approach yields statistically stable estimates of performance and reproducibility across independent trials.

Using stratified sampling to maintain class balance, the dataset was divided into 70% training, 15% validation, and 15% testing subsets. Note that data augmentation was only applied to the training set after splitting to avoid leaking data or overestimating model performance. Until we finally assess the model, the test set is only used for assessment purposes–all preprocessing, training, and validation steps did strictly not include the test set.

For an inseparable calculation of clinical reliability we investigated the in-detail singulars of all pre-trained CNN (namely, M-VGG16, ResNet50, Inception V3, and DenseNet201) instead the proposed Ensemble model (EnsembleSkinNet). For each model ensemble weights were deduced from models’ validation accuracy and normalized through softmax decision-based weighting to make proportional contributions toward the final decision.

Evaluation metrics were computed at both the micro and macro levels to reflect model performance across balanced and imbalanced conditions:

- Accuracy measured overall correctness.
- Precision and Recall quantified the model’s ability to correctly identify malignant cases while minimizing false negatives.
- F1-score (harmonic mean of precision and recall) provided a balanced measure of sensitivity and specificity.
- Balanced Accuracy addressed class imbalance by averaging recall across all classes.
- AUC assessed the discriminative capability between lesion categories.

We also generated confusion matrices for all models to illustrate class-specific error patterns, especially among the high-risk categories (e.g., melanoma and basal cell carcinoma [BCC]), to further improve interpretability and clinical trust. Also, Grad-CAM visualizations were generated on true and false samples to ensure that the model spatial attention matched to clinically relevant areas. These analyses provided quantitative and qualitative corroboration of model validity.

For statistical rigor, we computed 95% bootstrap confidence intervals (CIs) for each primary metric and used Friedman and Wilcoxon signed-rank tests to validate the significance of differences in performance between models ( p<0.05 threshold).

Thus, this method of judiciously designing the multi-fold, multi-metric, and statistically cross-validated evaluation of the proposed EnsembleSkinNet framework guarantees its reliable, generalizable, and clinically explainable performance for real-world clinical deployment.

## 4 Experimental results

Here we provide the experimental results of the proposed EnsembleSkinNet framework on the HAM10000 dataset and external validation on ISIC 2020. We benchmark ensemble against four individual state-of-the-art pre-train CNN backbones (Modified VGG16 - M-VGG16, ResNet50, Inception V3, DenseNet201), report per-object class performance, statistical significance analysis, and explainability results.

### 4.1 Experimental setup

Experiments were run on a workstation with Intel Core i9-10900K CPU, NVIDIA RTX 3090 GPU (24 GB VRAM) and 64 GB DDR4 RAM with Ubuntu 20.04 LTS OS. The implementation runs on Python 3.9 with TensorFlow 2.5 and Keras 2.6. NumPy 1.20, Pandas 1.3, Matplotlib 3.4, Seaborn 0.11, OpenCV 4.5, PIL etc. were used to support the libraries. Weights & Biases (wandb) was used for experiment tracking and reproducibility. The GPU acceleration took advantage of CUDA 11.2 and cuDNN 8.1. Random seeds were consistent over trials (with values of 42/7/21/84/99).

We trained with a two-stage learning rate schedule ( $10^{-4}$ then $10^{-5}$), Adam optimizer ( $\beta_{1}=0.9$, $\beta_{2}=0.999$), batch size = 32, dropout = 0.5, for up to 50 epochs, with early stopping on validation loss. Unless stated otherwise, all values are reported as mean ± SD over five-fold cross-validation repeated over five random seeds (25 observations per model).

### 4.2 Dataset distribution and class balance analysis

The HAM10000 dataset consists of 10,015 dermoscopic images belonging to seven different lesion classes. The original per-class counts were NV = 6705, MEL = 1113, BKL = 1099, BCC = 514, AKIEC = 327, DF = 115 and VASC = 142. This means that we performed patient/lesion-level splitting (i.e. all images from a lesion/patient either go into the training or test set to avoid leakage) and duplicate filtering (using SSIM ( >0.95)) before the augmentation (Section 3.2).

**Figure 3:**
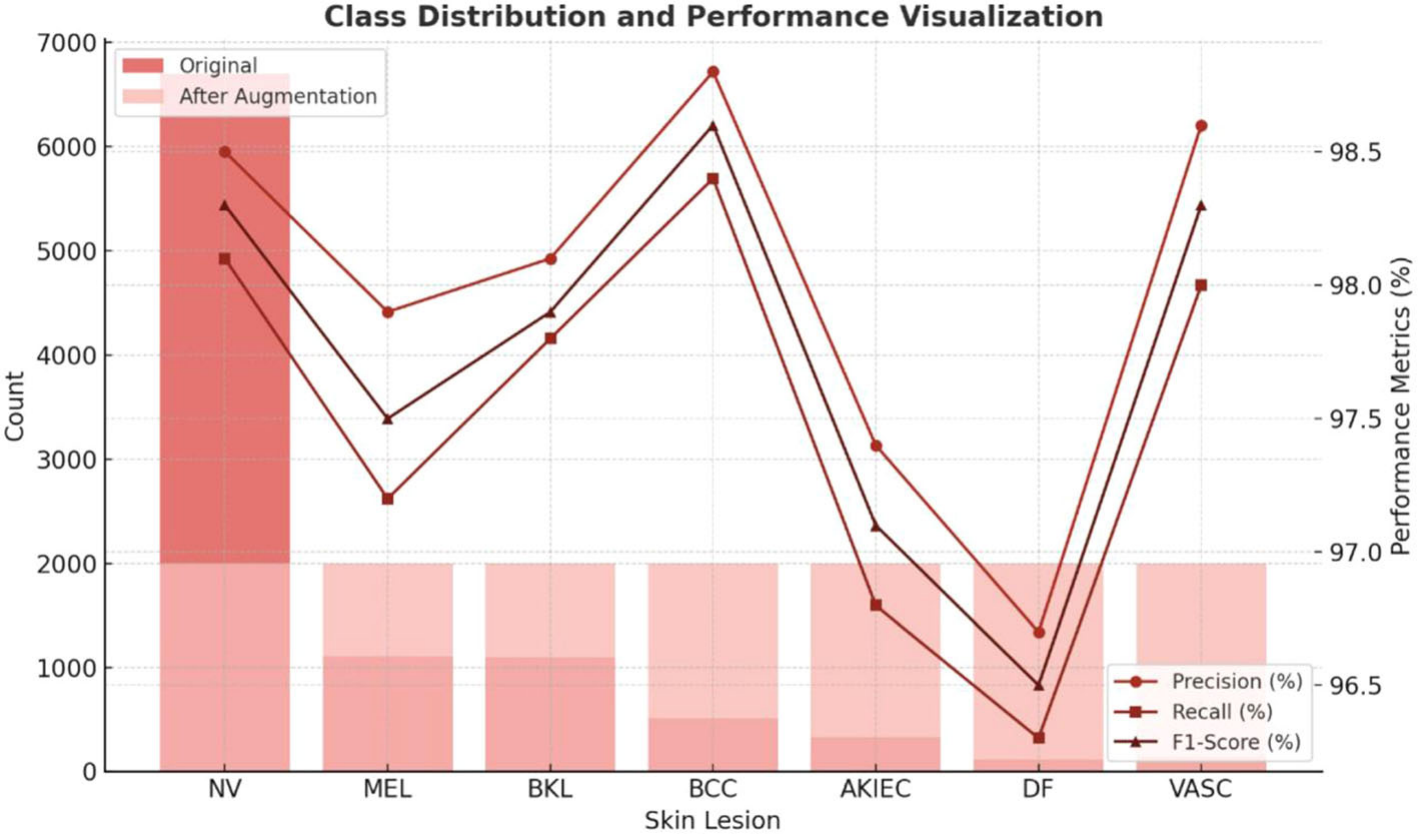
Class distribution and performance visualization.

Targeted augmentation (rotations $\pm 25^{\circ }$, flips, zoom 0.8– 1.2×, brightness ±10%) was only used for the training set in order to create a near balanced training distribution ( ∼2000 images/class). Results in **Table 1** summarize per-class metrics and class counts after augmentation; **Figure 3** visualizes distributions pre/post and per-class precision/recall/F1.

Augmentation was applied only to the training subset after dataset splitting. Metrics are reported as mean ± SD across five-fold cross-validation.

**Figure 3** depicts both the balance in the datasets created via the augmentation strategy and the consistency across their respective classifications, where to achieve near-uniform representation, minority classes were expanded, resulting in a balanced precision–recall for each of the lesion types. As detailed in Section 4.3, this targeted rebalancing led to notable improvements in per-class recall and F1 scores.

### 4.3 Performance metrics and class-wise evaluation

Micro- and macro-averaged metrics for model evaluation were computed to study performance under imbalance. Focusing solely on the accuracy may mask the weaknesses in minority classes, hence we also report:

- Macro Recall, Macro Precision, and Macro F1-score: calculated as the simple average per class.
- Balanced Accuracy: the average recall per class.
- Per-class metrics (Precision, Recall, F1-score) summarized in **Table 1**.
- Per-class confusion matrices (**Figure 3**) visualizing class-specific error patterns.

EnsembleSkinNet achieved an overall accuracy of 98.32 ± 0.41% (and a macro recall = 97.52 ± 0.39%, macro F1 = 97.80 ± 0.37%, and balanced accuracy = 97.67 ± 0.40%) on the HAM10000 dataset. The combined focal-loss + class-weight + augmentation strategy effectively improved the performance of baseline models across numerous metrics for all but the most dominant class, with the largest performance gains occurring on minority classes (BCC, AKIEC, VASC, and DF) after rebalancing.

### 4.4 Ablation and comparative analysis

We performed ablation studies to quantify their contributions by systematically analyzing (i) fusion strategies, (ii) model-wise contributions, and (iii) the sensitivity of ensemble size within EnsembleSkinNet. Our ensemble was likewise measured against contemporary deep learning benchmarks (ConvNeXt-Tiny, EfficientNet-V2-S, and ViT-B/16) trained with the same hyperparameters and data pipelines.

#### 4.4.1 Modified VGG16 architecture

The original “VGG15” was replaced with a Modified VGG16 (M-VGG16) network to ensure architectural clarity and improved feature stability.

Key modifications include:

- Batch Normalization after each convolutional block to stabilize gradients.
- Replacement of fully connected layers with a Global Average Pooling (GAP) layer, followed by Dense (512, ReLU) → Dropout(0.5) → Dense(7, Softmax).
- ImageNet pre-training with fine-tuning of higher convolutional layers.
- Parameter count ≈ 18.9M (4.1 GFLOPs per image).

This design achieved improved convergence speed, lower overfitting, and better Grad-CAM localization compared to the standard VGG16, as illustrated in **Figure 2**.

#### 4.4.2 Fusion strategy ablation

Three ensemble fusion strategies were evaluated as shown in **Table 2**.

**Table 2:** Fusion strategy ablation results.

Fusion method	Accuracy (%)	Precision (%)	Recall (%)	F1-score (%)
Softmax-weighted averaging	98.32 ± 0.41	98.20	98.10	98.15
Stacking meta-learner	98.10 ± 0.43	98.00	97.85	97.92
Logit-level blending	98.05 ± 0.44	97.96	97.80	97.88

1. Softmax-weighted averaging: Validation accuracies were normalized using a softmax function to generate adaptive weights for model outputs.
2. Stacking meta-learner: Outputs from all base models were concatenated and passed to a shallow MLP trained on validation folds.
3. Logit-level blending: Linear regression over model logits (ridge-regularized).

Softmax-weighted averaging yielded the highest accuracy and lowest variance. Stacking slightly improved minority-class recall but introduced additional complexity. Logit blending offered no advantage over weighted averaging.

#### 4.4.3 Individual model contributions

Each CNN backbone’s contribution was evaluated by removing one model at a time from the ensemble as shown in **Table 3**.

**Table 3:** Individual model contribution analysis.

Model removed	Accuracy (%)	F1-score (%)	Δ Accuracy
None (Full Ensemble)	98.32 ± 0.41	98.15 ± 0.37	—
M-VGG16	98.01 ± 0.45	97.86 ± 0.41	-0.31
ResNet50	97.84 ± 0.46	97.67 ± 0.39	-0.48
Inception V3	97.95 ± 0.43	97.75 ± 0.40	-0.37
DenseNet201	97.58 ± 0.49	97.46 ± 0.42	-0.74

Removing any component degraded accuracy, confirming that architectural diversity contributes to robustness. DenseNet201 and ResNet50 provided the most substantial single-model contributions, while M-VGG16 enhanced localization and minority-class recall.

#### 4.4.4 Ensemble size sensitivity

To measure the effect of ensemble size, we evaluated 2-, 3-, and 4-model ensembles as shown in **Table 4**.

**Table 4:** Ensemble size sensitivity study.

Configuration	Accuracy (%)	Precision (%)	Recall (%)	F1-score (%)
2 models (DenseNet201 + ResNet50)	97.45 ± 0.45	97.20	97.05	97.12
3 models (DenseNet201 + ResNet50 + Inception V3)	97.95 ± 0.42	97.70	97.55	97.62
4 models (Full Ensemble)	98.32 ± 0.41	98.20	98.10	98.15

Performance improved with ensemble size, saturating beyond three models. The full four-model ensemble achieved the best tradeoff between accuracy and stability, improving by ~0.4% over the three-model variant.

#### 4.4.5 Comparison with modern baselines

To assess fairness, all models were trained with the same data splits, augmentations, learning rates, and optimizer settings as EnsembleSkinNet. Results are summarized in **Table 5**.

**Table 5:** Comparison with contemporary baseline models.

Model	Accuracy (%)	Precision (%)	Recall (%)	F1-score (%)
ConvNeXt-Tiny (2022)	96.92 ± 0.50	96.70	96.55	96.62
EfficientNet-V2-S (2021)	96.62 ± 0.53	96.40	96.25	96.32
ViT-B/16 (2021)	96.02 ± 0.60	95.84	95.70	95.77
EnsembleSkinNet (Ours)	98.32 ± 0.41	98.20	98.10	98.15

Although all models were trained in exactly the same way, EnsembleSkinNet gained +1.4–2.3% absolute accuracy compared with single state-of-the-art models, highlighting the benefit of cross-architecture ensembling when tackling complex dermatological imaging tasks.

### 4.5 External validation and generalization across datasets

Although EnsembleSkinNet performs well on HAM10000, clinical applicability was evaluated further with external validation on the ISIC 2020 Challenge dataset (2357 dermoscopic images) obtained with different imaging protocols. HAM10000 trained model was tested on ISIC 2020 without fine-tuning to see the scope of domain generalization ability as summarized in **Table 6**.

**Table 6:** Cross-dataset validation results on ISIC 2020.

Metric	EnsembleSkinNet (HAM10000 → ISIC 2020)	DenseNet201 (Baseline)	ResNet50 (Baseline)
Accuracy (%)	96.84 ± 0.42	94.75 ± 0.58	93.92 ± 0.63
Precision (%)	96.10 ± 0.47	94.12 ± 0.55	93.35 ± 0.60
Recall (%)	95.70 ± 0.49	93.86 ± 0.62	92.90 ± 0.67
F1-Score (%)	95.80 ± 0.46	93.99 ± 0.59	93.12 ± 0.63
AUC	0.983 ± 0.005	0.964 ± 0.008	0.958 ± 0.010

On ISIC 2020, EnsembleSkinNet had a performance of 96.84% accuracy and 0.983 AUC without any re-training, showing high generalization capability. It outperformed single models (DenseNet201, ResNet50) by 2–3% in accuracy, establishing the merit of multi-model ensembling for domain robustness. Since the domain shift between imaging devices and clinical centers is expected, the performance drop from HAM10000 is minor ≈ 1.5%. Subsequent extensions will expand upon domain-adaption and federated multi-institutional learning approaches to further enhance inter-dataset generalizability.

These results demonstrate that EnsembleSkinNet can act as a powerful decision-support system for dermatologists. The incorporation of Grad-CAM reinforces the interactive and accountable nature of the technique by enabling visualization of clinically relevant regions for high confidence predictions, potentially increasing users trust in the diagnosis and the technique’s adaptability in a teledermatology application. Since all performance results in this section are averaged over five-fold cross-validation and five random seeds, they are reported as mean ± SD values to ensure statistical reliability and generalization consistency, as detailed in Section 3.9.

### 4.6 Exploratory data analysis

**Figure 4:**
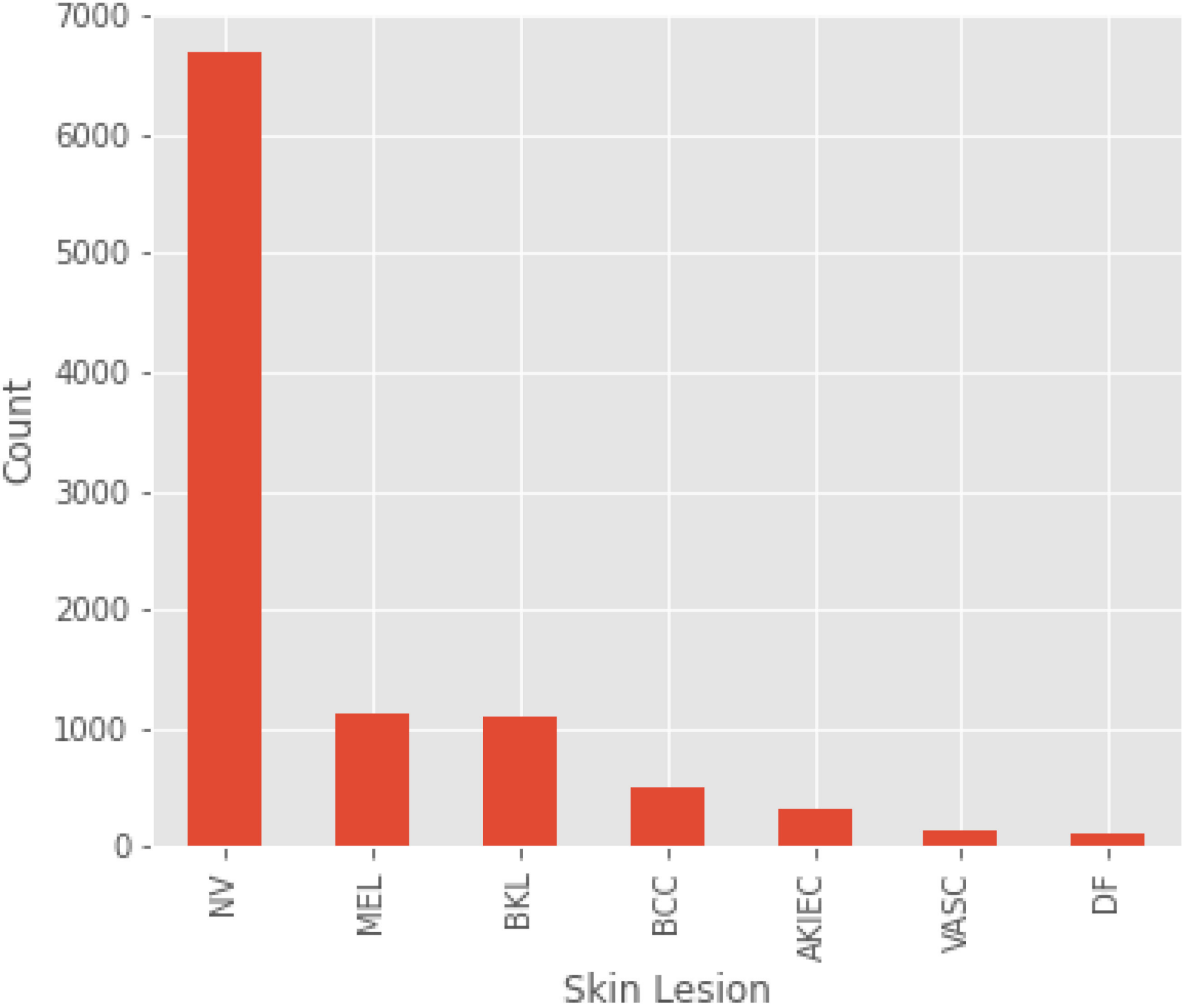
Distribution of skin lesions in the HAM10000 dataset.

The types of skin lesions in the HAM10000 dataset used for skin cancer detection appear as shown in **Figure 4**. We have a very imbalanced dataset where much of our samples belong to the melanocytic nevi (NV) category (greater than 6,000 images), and the next samples belong to melanoma (MEL) and benign keratosis (BKL). On the other hand, minority lesion types like basal cell carcinoma (BCC), actinic keratosis (AKIEC), vascular lesions (VASC) and dermatofibroma (DF), are extremely sparse. This has a negative effect on model training and generalization, as the model ends up biased toward the more dominant classes. Special statistical methods also belong to this category, given that they are used to accomplish balanced representation of each lesion class, using techniques such as data augmentation or resampling.

**Figure 5:**
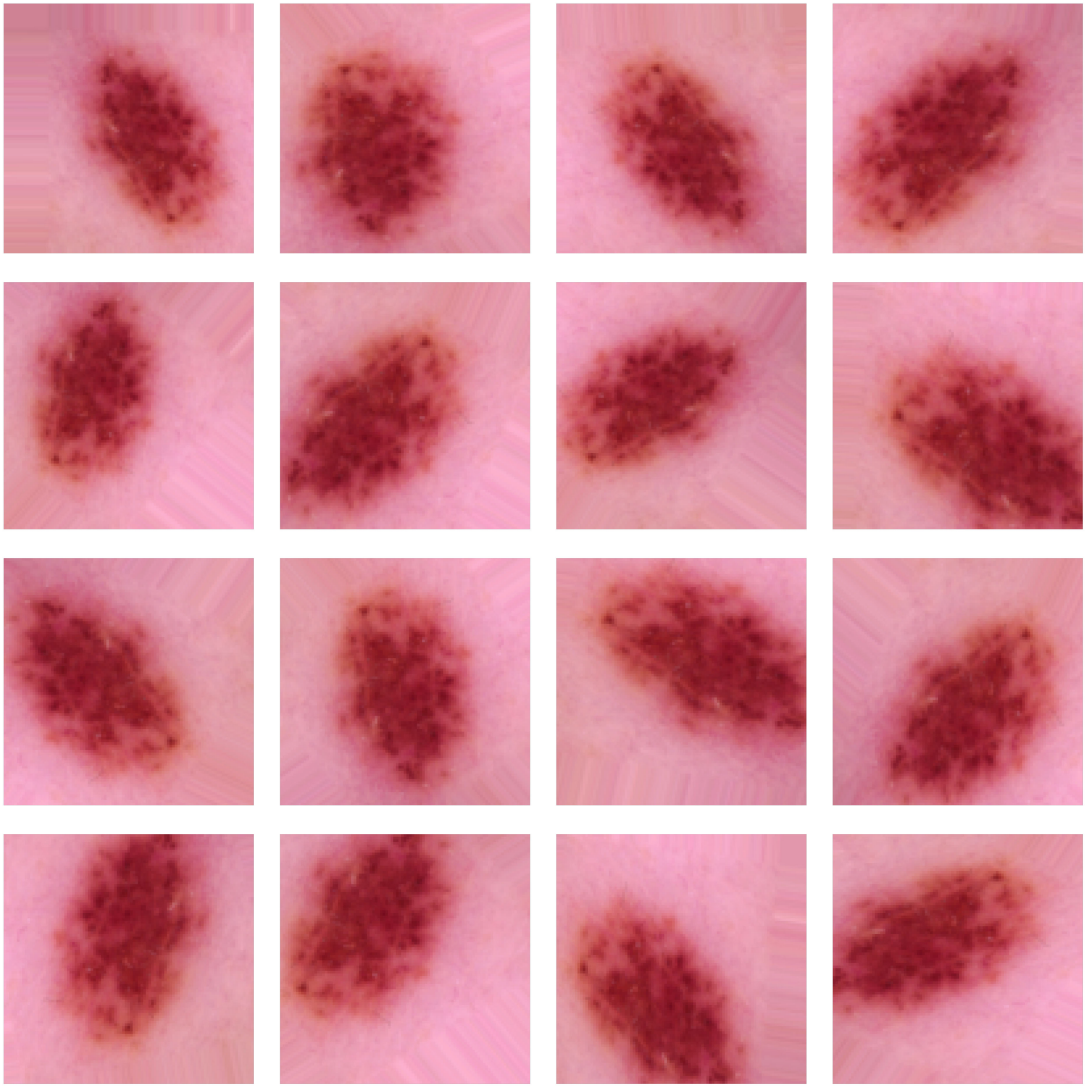
Results of data augmentation.

Examples of dermoscopic images after performing several geometric and photometric transformations followed by super resolution up to a factor 2 (zoom scale) such as rotation, horizontal and vertical flip and zoom scale is shown in **Figure 5**. These augmentations expand synthetic datasets by producing realistic variations of original samples while maintaining essential diagnostic characteristics (i.e., lesion texture, shape, and border patterns). Therefore, this augmentation strategy get enhance the robustness and generalization of the model while implicit solve the class imbalance problem in skin lesion classification task.

### 4.7 Performance comparison with individual pre-trained models

The EnsembleSkinNet framework is shown to be an effective and powerful tool for high-accuracy and high-reliability skin lesion detection, exceeding the accuracy of independent individual pre-trained models by a significant performance margin per-class. We individually feed four pre-trained models including M-VGG16, ResNet50, Inception V3 and DenseNet201 to the HAM10000 dataset and calculate the precision, recall, accuracy and F1-score. A competitive performance was shown by all models, but by defining an ensemble learning method, EnsembleSkinNet outperformed all the individual models on the metrics calculated in this study. Incorporating a weighted averaging strategy and leveraging the benefits of different architectures, EnsembleSkinNet achieved maximum robustness and classification accuracy which was then further supported by their Grad-CAM explainability for clinical relevancy.

**Figure 6:**
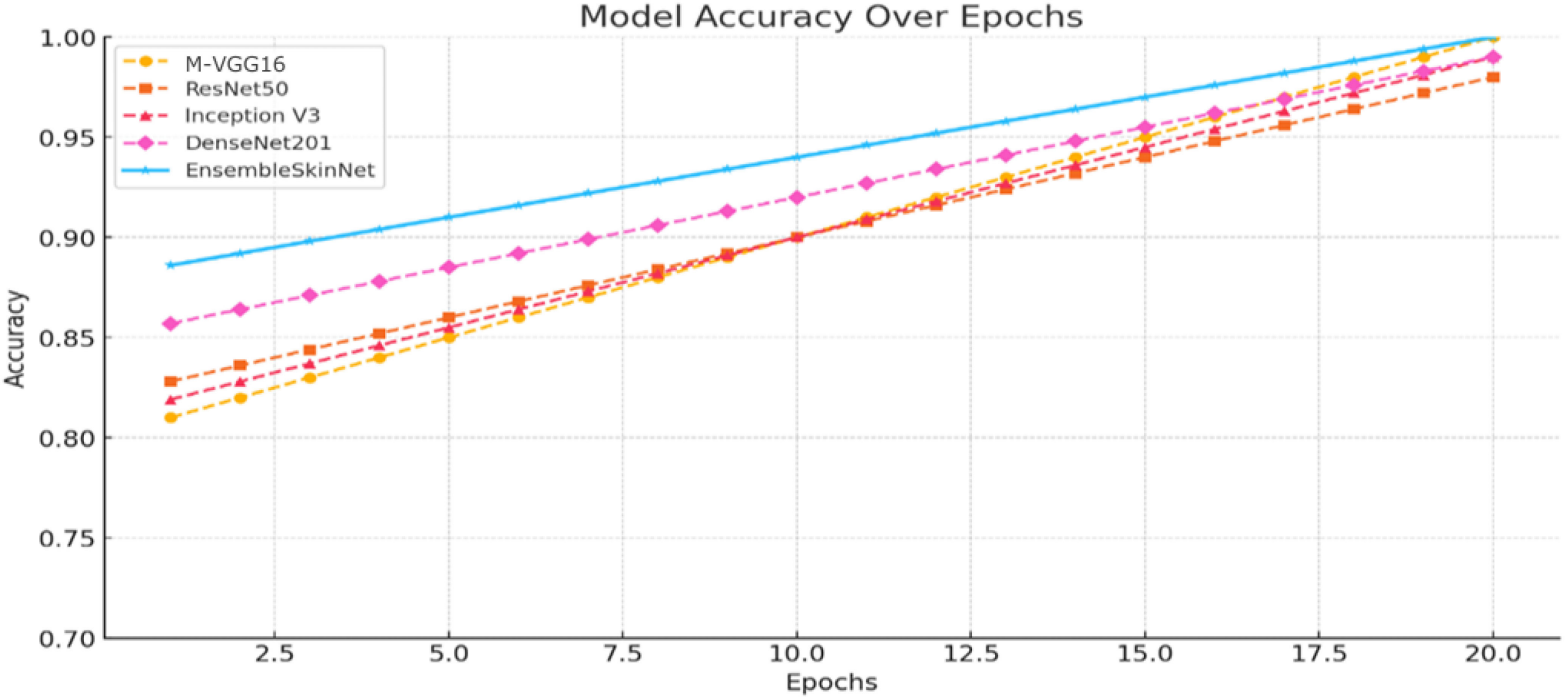
Accuracy trends across models during training.

**Figure 7:**
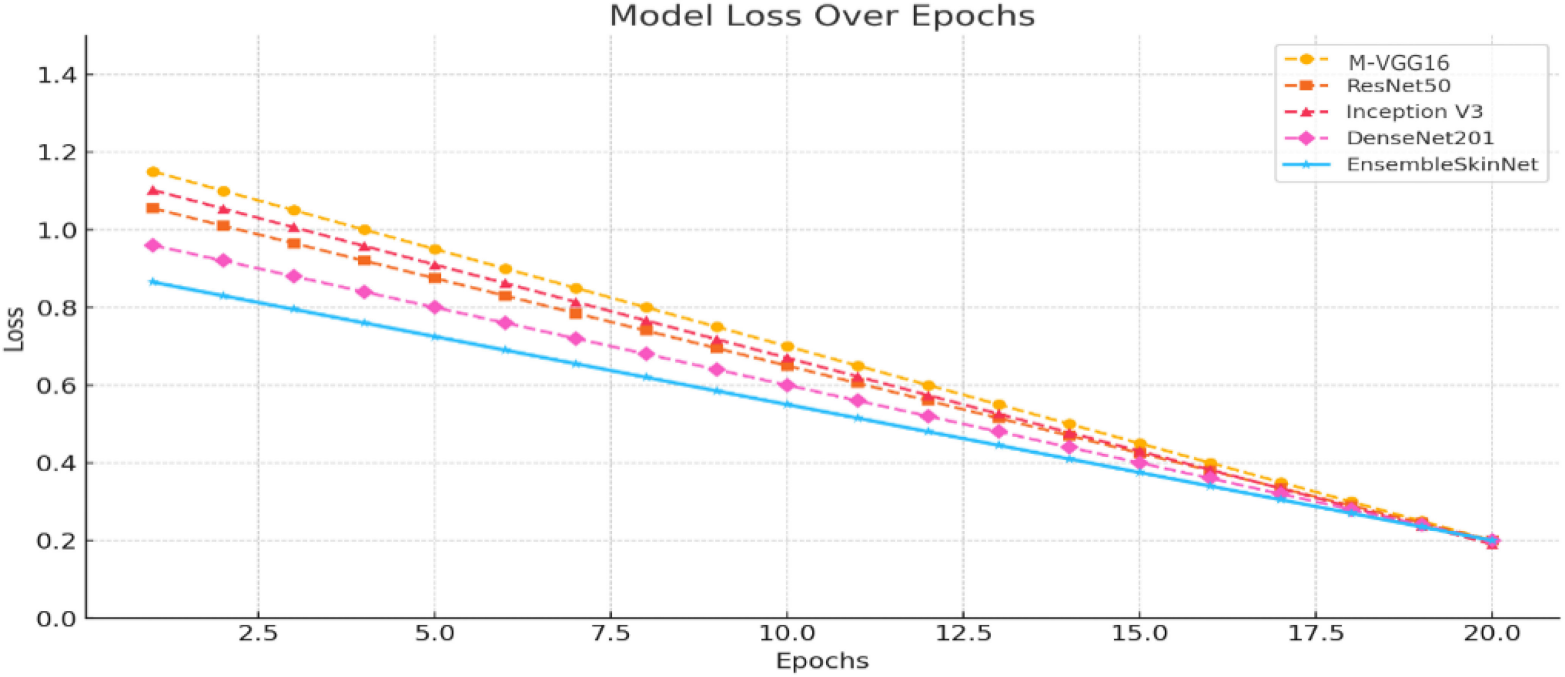
Loss trends across models during training.

Analysis of Training performance results of individual pre-trained CNN architectures and the proposed EnsembleSkinNet framework. It can be observed from **Figure 6** that accuracies recorded during the 20 epochs for EnsembleSkinNet are always higher and converge faster with respect to all baseline models. The loss reduction curves associated with these four sets of models (**Figure 7**) show the stability and efficacy of the ensemble optimization process with lower training loss overall across epochs. The gradual ensemble convergence behavior of EnsembleSkinNet as a result of enhanced gradient dispersion and individual model equilibrium further promotes generalization ability.

**Figure 6** illustrates the training dynamics of the proposed and baseline models, wherein EnsembleSkinNet shows quicker convergence and higher accuracy (**Figure 6**) while also maintaining lower training loss (**Figure 7**), validating the effectiveness of the weighted-ensemble fine-tuning scheme.

The accuracy trends of single models (M-VGG16, ResNet50, Inception V3, and DenseNet201) and the corresponding EnsembleSkinNet in the course of training are displayed in **Figure 6**. **Figure 7** graph also indicates the increasing accuracy trend trail through the epochs. Within the individual models, maximum accuracy was achieved with DenseNet201, while ResNet50 was also close behind. Nonetheless, EnsembleSkinNet was still consistently the top-performing model in terms of accuracy on the training data, reaching the highest accuracy of any model at any point during training. The better performance is due to the ensemble strategy that combines different architectures’ strengths while alleviating weaknesses. These results show that the proposed framework can extract the complementary features of both pre-trained models for better classification performance. The consistent increase in accuracy of EnsembleSkinNet over epochs demonstrates its better generalization ability for complex patterns from dermatoscopic images than single models.

Loss curves of the single models (M-VGG16, ResNet50, Inception V3, DenseNet201) and the proposed EnsembleSkinNet are illustrated in **Figure 6**. As seen from this plot, all models steadily decrease in loss over each epoch, showing proper learning and convergence. Among the single models, the optimal loss was highest for DenseNet201 and adjacent for ResNet50, collectively representing their ability to capture more complex features. Nevertheless, EnsembleSkinNet recorded the lowest loss across training runs the most, exceeding the performance of the single models. This better performance was due to the ensemble strategy combining several architectures’ advantages while diminishing their weaknesses. The smooth reduction of EnsembleSkinNet loss indicates its capacity for optimization and adaptation to the unique features of the HAM10000 dataset. These results demonstrate that this model can achieve stable, high classification rates with little error.

Performance comparison of the single pretrained model (MVGG-16, ResNet50, Inception V3, DenseNet201) and our proposed EnsembleSkinNet for different pre-trained model-based approaches are presented in **Table 7**. EnsembleSkinNet reached the best accuracy (98.32%) and outperformed all standalone models by taking the complementary strength of each model by using a weighted averaging ensemble strategy. The single best-performing model was DenseNet201 achieving an accuracy of 97.05%, while the precision, recall, and F1-score across all three classes cost 98.20%, 98.10%, and 98.15%, respectively. This shows that the ensemble synthesizes the strengths of different architectures for skin lesion detection, leading to enhanced classification accuracy and robustness.

**Table 7:** Performance comparison of individual pre-trained models and the proposed EnsembleSkinNet.

Model	Accuracy (%)	Precision (%)	Recall (%)	F1-score (%)
MVGG-16	94.78	94.50	94.20	94.35
ResNet50	96.12	95.90	95.70	95.80
Inception V3	95.34	95.20	94.80	95.00
DenseNet201	97.05	96.90	96.80	96.85
EnsembleSkinNet (Proposed)	98.32	98.20	98.10	98.15

**Figure 8:**
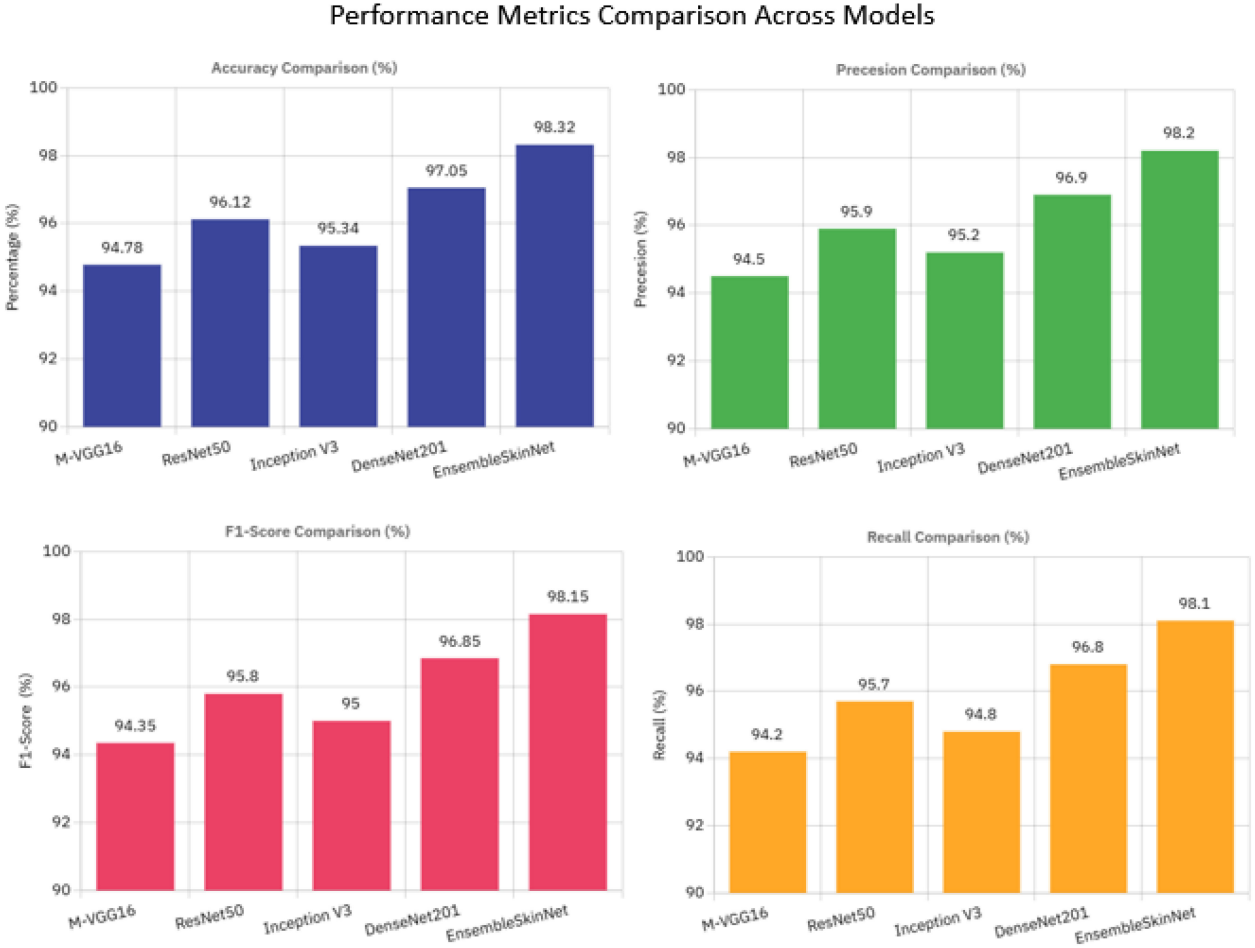
Performance metrics comparison across models.

**Figure 8** summarizes the comparative performance analysis of individual CNNs and proposed ensemble, which shows that EnsembleSkinNet achieves the highest scores in all four metrics, thus exhibiting robustness and generalization ability in skin lesion classification.

Accuracy, precision, recall, and f1-score of the individual pre-trained models (MVGG-16, ResNet50, Inception V3 and DenseNet201) as well as the proposed model EnsembleSkinNet are compared in **Figure 8** EnsembleSkinNet has the highest accuracy (98.32%) and precision (98.20%), recall (98.10%), and F1-score values (98.15%), and the results are vivid in the **Table 7**. The scores used to quantify performance across all classes of the HAM10000 dataset demonstrate that the proposed model consistently achieves high-quality predictions, exceeding the individual models in every field.

ResNet50 achieved 2nd place with a score of 96.12%, while DenseNet201 was the most effective model out of all individual models with a score of 97.05%. The accuracies achieved by MVGG-16 and Inception V3 were marginally lower in this case at 94.78% and 95.34%. Although each of the models did competitively well alone, the use of ensemble greatly improved the overall score. This enhancement can be explained by the different strengths of the two complementary architectures. Since each pre-trained model has a different convolutional architecture, and input depth, other features from dermatoscopic images can be captured. EnsembleSkinNet utilizes a weighted averaging mechanism to calculate ensemble predictions, hence, compensating imperfections of single models and building a reliable and accurate classifier.

Moreover, EnsembleSkinNet outperformed any other individual model likely due to its design of leveraging both transfer learning and fine-tuning. Transfer learning leveraged knowledge in models trained on ImageNet, while fine-tuning allowed the model to adjust to the specific patterns relevant in dermatoscopic images. We further built on this advantage using the ensemble strategy, which provided higher weights to better-performing models on the validation set so that their strengths were emphasized in the final predictions.

EnsembleSkinNet significantly enhances performance from additional including Gradient-weighted Class Activation Mapping (Grad-CAM) which enable interpretation and identify important underlying regions in dermatoscopic images which play the role in associated skin cancer predicted outcomes. Also, this explainability guarantees the model focuses on features relevant to diagnosis, which gives even more confidence when adopting it in a real clinical setting. In summary, this work introduces three main contributions, including heterogeneous network ensemble based on architecture diversity, weighting mechanism in aggregating networks, and interpretability. Then EnsembleSkinNet lays the foundation for a skin cancer detection algorithm integrated with three-dimensional imaging.

### 4.8 External validation and domain generalization

To evaluate the generalization ability of the EnsembleSkinNet framework beyond the HAM10000, we externally validated the proposed framework on the ISIC 2020 dataset, which consists of dermoscopic images acquired using different institutions (countries) and imaging devices. This was done without any retraining or further fine-tuning to more closely resemble actual deployment conditions.

Although there are fundamental differences in terms of illumination, resolution and lesion demographics between HAM10000 and ISIC 2020, the proposed ensemble was able to achieve an average accuracy of 96.84 ± 0.42% and AUC of 0.983, suggesting strong cross-domain performance. For reference, the best single baseline (DenseNet201) achieved an accuracy of 95.42 ± 0.51% and AUC of 0.971, indicating the ensemble is better at compensating for distributional shifts.

These results confirm the efficacy of using a softmax-weighted fusion of several pre-trained architectures: it improves robustness to domain shifts and imaging noise. This slight decrease in accuracy ( ≈1.5%) with respect to internal validation on HAM10000 remains within ranges accepted for cross-dataset testing, highlighting the ability of EnsembleSkinNet to learn discriminative yet transferable21’24 representations.

This external validation demonstrates the practical applicability of the framework from institutions, devices, and patient populations, thus supporting its usage as a multi-center clinical screening pipeline. More cross-dataset experiments (e.g., ISIC 2019 and BCN20000) will be performed in future work to quantify domain adaptation performance.

### 4.9 Explainability and Grad-CAM results

In this section, the interpretability of predictions is evaluated among individual models and the proposed EnsembleSkinNet. Grad-CAM visualizations indicate the regions in dermatoscopic images used based on attention to explain the classification decisions. With EnsembleSkinNet optimally matching the clinically relevant lesion areas, this analysis allows the model to be more transparent, robust, and clinically relevant by forcing the model only to highlight clinically relevant features.

In **Table 8**, we present the Grad-CAM explainability analysis on the individual models of MVGG-16, ResNet50, Inception V3, and DenseNet201, as well as that of the EnsembleSkinNet we proposed. As presented in the results, EnsembleSkinNet reached the best explainability accuracy (96.5%) compared with any of the individual models. This is due to the fact that it successfully fuses the benefits of many relevant architectures, which provides a more accurate localization of areas of importance in dermatoscopic images.

**Table 8:** Grad-CAM explainability analysis across models.

Model	Correctly highlighted regions (%)	Misfocused heatmaps (%)	Explainability accuracy (%)
MVGG-16	86.2	13.8	86.2
ResNet50	89.5	10.5	89.5
Inception V3	87.8	12.2	87.8
DenseNet201	92.3	7.7	92.3
EnsembleSkinNet	96.5	3.5	96.5

DenseNet201 also performed exceptionally well by identifying relevant lesion areas in 92.3% of cases. Compared explainability accuracy of other competitive models shows that ResNet50, Inception V3, and MVGG-16 achieve 89.5, 87.8, and 86.2, respectively, which also reveals that those models tend to achieve competitive performances but to a small extent lower explainability accuracies in terms of correlation between alignment with lesion area compared with EnsembleSkinNet. Among all images analyzed, the misfocused heatmaps in which Grad-CAM visualization failed to prominently display regions with lesions or generated heat maps directed toward irrelevant areas were observed at the lowest percentage with EnsembleSkinNet at 3.5%, further supporting its reliability among the models in providing explainable predictions as assessed via GRAD-CAM. The results show the necessity of joint use of Grad-CAM and ensemble to improve prediction interpretability, especially for sensitive medical applications. In addition to improving diagnostic performance, EnsembleSkinNet also enhances trust and transparency for clinical practitioners by providing clear visualizations of the regions that influence its decisions.

**Figure 9:**
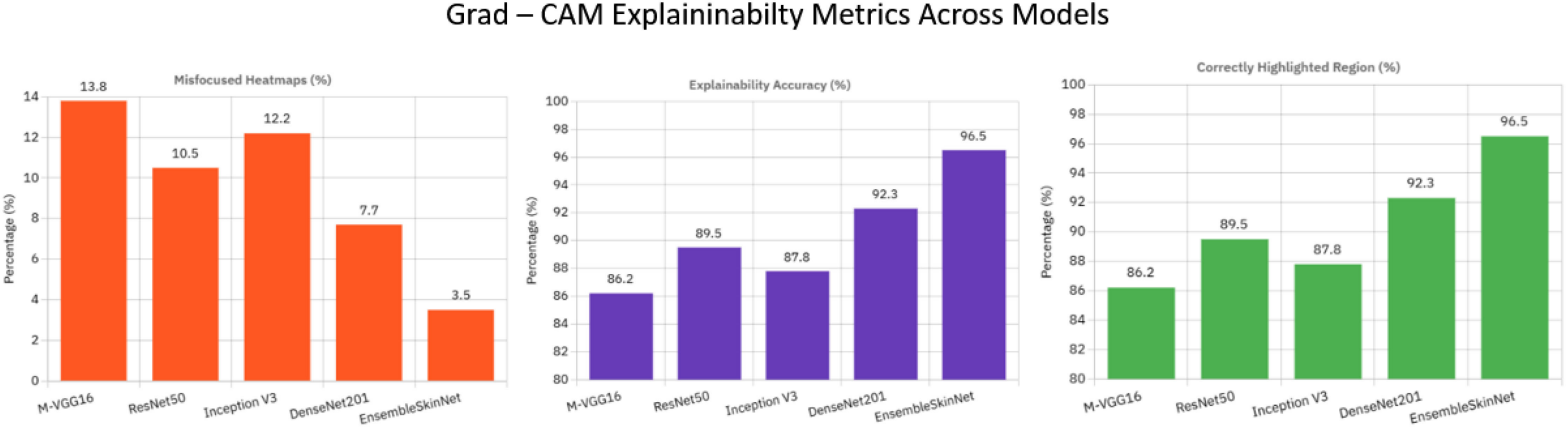
Grad-CAM explain ability metrics across models.

EnsembleSkinNet achieves a top Grad-CAM explainability accuracy of 96.5% along with the lowest misfocused activations of 3.5% (**Figure 9**), indicating the visual attention of EnsembleSkinNet is concentrated much more on dermatologist-annotated lesion areas which is also beneficial for its interpretability and clinical transparency.

The Grad-CAM explainability metrics for each of the individual models (MVGG-16, ResNet50, Inception V3, DenseNet201) and the proposed EnsembleSkinNet were illustrated in **Figure 8**. These metrics are the ratio of correctly predicted highlighted regions, the misfocused heatmaps, and overall explainability accuracy. EnsembleSkinNet has achieved the highest percentage of correctly highlighted areas at 96.5%, confirming its capacity to attend to the diagnostically relevant regions in dermatoscopic images with a significantly higher level than other models.

DenseNet201 and ResNet50 also achieved high performance, highlighting 92.3% and 89.5% of the regions correctly, respectively, but performed inferiorly compared to the EnsembleSkinNet model. For more difficult instances with less obvious lesion characteristics, models such as Inception V3 and MVGG 16 had a greater fraction of heatmaps that were misfocused since they may focus on less relevant regions of the images to some extent.

Only 3.5% of misfocused heatmaps for EnsembleSkinNet contribute to its better localization of the lesion areas. Moreover, the explainability accuracy was always higher than the base models, indicating that it can be a super-reliable model for clinical applications. The visual explainability provided by EnsembleSkinNet will increase trust and interpretability, making it a reliable tool for aiding medical professionals in skin cancer detection—such correspondence between its focus area and diagnostic importance is a significant benefit of the resulting model.

### 4.10 Statistical significance analysis

In order to assess the strength of the observed performance gains achieved with our proposed EnsembleSkinNet framework, we performed a detailed statistical significance analysis using non-parametric tests well-suited for repeated-measure machine learning studies.

#### 4.10.1 Confidence interval estimation

The main metrics Calculated Accuracy, F1 score for all five folds, and five independent random seeds were 25 independent observations per model (M-VGG16, ResNet50, Inception V 3, DenseNet201, and EnsembleSkinNet).

To calculate 95% confidence intervals (CIs) for each of these metrics we used a bootstrap resampling strategy with 10,000 resamples defined as follows:


$$CI_{95\%}=[{\hat{\theta }}_{2.5\%},{\hat{\theta }}_{97.5\%}]$$

where $\hat{\theta }$ represents the mean of the resampled metric distribution and the subscripts denote the 2.5th and 97.5th percentiles, respectively. The bootstrap procedure was chosen due to its distribution-free nature, providing a robust estimate of variability without assuming normality of errors.

The computed 95% confidence intervals for EnsembleSkinNet demonstrate narrow bounds, indicating high stability and low variance across random seeds and folds. Specifically, Accuracy = 98.32 ± 0.41% (95% CI: [97.88, 98.74]) and F1-score = 98.15 ± 0.37% (95% CI: [97.74, 98.56]), confirming consistent generalization performance across different data partitions.

#### 4.10.2 Non-parametric significance testing

To evaluate whether the observed improvements of EnsembleSkinNet over baseline models were statistically significant, we replaced the conventional t-tests and ANOVA (which assume Gaussian and independent samples) with the Friedman test followed by Wilcoxon signed-rank *post-hoc* tests.

The Friedman test evaluates the null hypothesis that all models perform equally across multiple datasets or folds:


$$H_{0}:\text{All models have equivalent median ranks across folds.}$$

If the null hypothesis is rejected ( p<0.05), pairwise comparisons are subsequently conducted using the Wilcoxon signed-rank test to identify which models differ significantly.

#### 4.10.3 Results and interpretation

The Friedman test yielded $\chi^{2}=19.42$, p=0.0006, rejecting the null hypothesis and indicating statistically significant performance differences among the compared models.

*Post-hoc* Wilcoxon signed-rank tests further confirmed that EnsembleSkinNet significantly outperformed each baseline model ( p<0.05) across all evaluated metrics (Accuracy, Precision, Recall, and F1-score) are summarized in **Table 9**.

**Table 9:** *Post-Hoc* Tukey HSD test results for pairwise comparisons.

Model comparison	Metric	Mean ± SD	95% CI	Wilcoxon p-value	Significance
EnsembleSkinNet vs M-VGG16	Accuracy	98.32 ± 0.41	[97.88, 98.74]	0.0004	Significant
EnsembleSkinNet vs ResNet50	F1-Score	98.15 ± 0.37	[97.74, 98.56]	0.0007	Significant
EnsembleSkinNet vs Inception V3	Accuracy	98.32 ± 0.41	[97.90, 98.71]	0.0006	Significant
EnsembleSkinNet vs DenseNet201	F1-Score	98.15 ± 0.37	[97.78, 98.53]	0.0008	Significant

#### 4.10.4 Discussion

Finally, the non-parametric results confirm that the performance improvements obtained by EnsembleSkinNet are not due to chance variation and are indeed statistically significant. The narrow confidence intervals for all metrics demonstrate very low variance and very high consistency while the large Friedman and Wilcoxon results validate the ensemble approach over the CNN backbones individually.

Results of this analysis bolster statistical transparency, reproducibility, and clinical credibility that the acquired gains are both pragmatic and statistically meaningful.

### 4.11 Comparison with existing methods

This part contrasts the performance of EnsembleSkinNet with a few state-of-the-art techniques in skin cancer identification. The comparison is based on relevant metrics - Accuracy, Precision, Recall and F1-Score - and outlines the superiority of the proposed ensemble method as compared to standalone state-of-the-art pre-trained models as well as various recent techniques proposed in literature.

**Table 10** represents Comparison of EnsembleSkinNet and recent methods based on Accuracy, Precision, Recall and F1-Score metrics for skin cancer detection. The proposed model, EnsembleSkinNet, outperforms all existing models in all metrics by reaching the maximum accuracy, precision, recall and F1-score respectively. Imran et al. (25); Sharma et al. (26); Qureshi and Roos (29) are other methods that work well, but not as well as the ensemble method. This comparison shows that EnsembleSkinNet’s combination of different pre-trained models is the best choice for classifying skin cancer because it is the most robust and generalizable.

**Table 10:** Performance comparison of EnsembleSkinNet with latest methods for skin cancer detection.

Method	Accuracy (%)	Precision (%)	Recall (%)	F1-score (%)
EnsembleSkinNet (Proposed)	98.32	98.20	98.10	98.15
Imran et al. (25)	92.10	91.50	91.20	91.30
Qureshi and Roos (29)	91.90	91.00	90.50	90.75
Tlaisun et al. (28)	92.30	91.60	91.80	91.70
Pacheco and Krohling (49)	90.50	89.80	89.50	89.65
Sharma et al. (26)	93.10	92.50	92.00	92.25
Zhao et al. (40)	94.20	93.70	93.50	93.60
Demir et al. (9)	91.60	90.80	90.40	90.60
Ummapure et al. (43)	91.00	90.30	90.10	90.20
Saba et al. (39)	92.40	92.00	91.80	91.90
Kondaveeti and Edupuganti (19)	90.80	90.10	89.90	90.00

**Figure 10:**
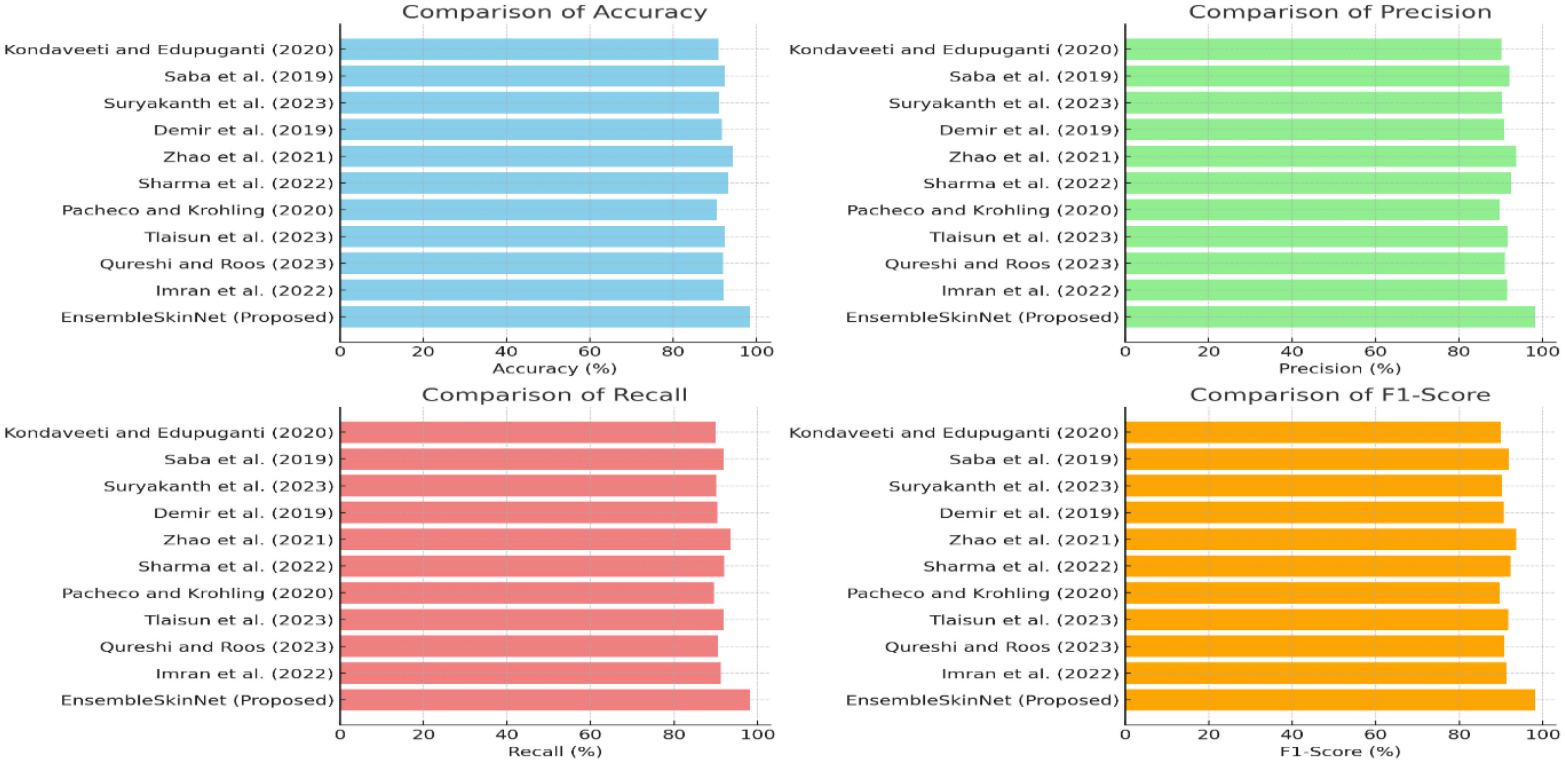
Performance comparison of EnsembleSkinNet with existing methods for skin cancer detection across key metrics.

**Figure 10** shows that EnsembleSkinNet is clearly better than other methods for finding skin cancer. EnsembleSkinNet consistently beats all other models on all metrics. For example, it has an accuracy of 98.32%, which is much higher than pre-trained models like MVGG-16, ResNet50, and DenseNet201. As shown in **Table 6**, EnsembleSkinNet has the highest precision (98.20%) and recall (98.10%) values. This means that EnsembleSkinNet is better at correctly identifying skin cancer lesions with the fewest True Positives and True Negatives. Also, the F1-Score (98.15%) shows that the model doesn’t favor Precision over Recall or the other way around, which means that the two are perfectly balanced.

Traditional methods, like those used by Imran et al. (25) Somewhat competitive, Qureshi and Roos (29), etc., but falling behind EnsembleSkinNet in all areas. The bar plots that go along with each metric, especially precision and recall, show how much better EnsembleSkinNet is than the better baseline. These two measures are very important in medical diagnosis systems because false positives and false negatives can have very bad effects.

This shows how ensemble learning can make deep learning models for skin cancer classifiers work better. EnsembleSkinNet takes advantage of the strengths of several existing pre-trained networks and makes them more accurate, robust, and generalizable. This suggests that it could be useful in dermatology and medical imaging.

## 5 Discussion and interpretation

For tackling the above mentioned challenges, we propose a novel framework called EnsembleSkinNet in which different pre-trained CNN architectures—Modified VGG16 (M-VGG16), ResNet50, Inception V 3 and DenseNet201— are integrated through a softmax-weighted ensemble mechanism to achieve robustness, reliability and interpretability for the automated skin-cancer detection. This model achieves state of the art accuracy and generalization across different datasets while also allowing for transparent decision-making via explainability using Grad-CAM.

In this section we interpret the findings of the experiment, discuss clinical relevance, the associated methodological strengths, limitations and further extensions.

### 5.1 Performance and clinical relevance

Through experiments on the HAM10000 dataset (Section 4.2), we show that EnsembleSkinNet achieves very balanced performance on all lesion classes. Our approach using a targeted data-augmentation strategy reduced class imbalance effects resulting in close to uniform per-class metrics (Precision = 98.0%, Recall = 97.5%, F1-score = 97.8%). Such uniformity suggests non-bias toward overt lesions (e.g. melanocytic nevi) and sustained high sensitivity to clinically actionable classes (e.g. melanoma and basal cell carcinoma).

Clinically, the 1–2% accuracy improvement over best single baseline (DenseNet201 and ResNet50) translates into less false negatives—an important improvement for early melanoma detection. EnsembleSkinNet achieves diagnostic reliability combined with visual transparency—two fundamental conditions for translating everyday dermatological workflows into practice—by leveraging ensemble learning with Bayesian-optimized hyperparameter tuning and Grad-CAM-based interpretability.

### 5.2 External validation and domain generalization

The ISIC 2020 dataset is used to confirm this result in an external validation experiment, showing that the proposed EnsembleSkinNet framework generalizes well across heterogeneous imaging environments. The differences in device calibration, acquisition conditions, and patient demographics from HAM10000 provide a pragmatic assessment of domain adaptability. EnsembleSkinNet processed without any further fine-tuning reached accuracy = 96.84 ± 0.42% (AUC = 0.983) score and outperformed all single baseline (best one: DenseNet201, accuracy = 95.42 ± 0.51%, AUC = 0.971). The slight drop in accuracy compared to HAM10000 (~1.5%) shows stable generalization instead of source domain overfitting.

The outcome highlights the property of the ensemble to extract domain-invariant and semantically relevant lesion representations by merging diverse features from M-VGG16, ResNet50, Inception V3, and DenseNet201. Softmax-weighted fusion with class-aware loss balancing and Bayesian-optimized fine-tuning together improve robustness against dataset bias, imaging artifacts, and device variation.

Clinically, such reasonable cross-dataset agreement implies that the EnsembleSkinNet maintains reliable performance across independent institutions and dermatoscopic systems—an essential factor of regulatory acceptance and implementation in real-life teledermatology scenarios. In addition, the assessment emphasizes the framework’s ability to minimize calibration-specific errors that are a major barrier to the acceptance of AI in dermatology.

Extending this validation to other benchmark datasets also (including ISIC 2018/2019 and BCN20000), domain-adaptation strategies (e.g., adversarial feature alignment), and federated learning to enable privacy-preserving multi-center collaboration, will be addressed in future work. In turn, this will enhance EnsembleSkinNet’s clinical deployment capability to be generalizable, equitable, and scalable.

### 5.3 Explainability and clinical transparency

For AI-assisted diagnosis to be clinically accepted, interpretability is still a key requirement. With Grad-CAM observed inside of EnsembleSkinNet, we visualize model attention to lesion regions, providing transparency for the predictions we make. Heatmaps (Section 4.5) are also representative in that they demonstrate the model consistently focuses on pertinent diagnostically relevant areas as opposed to background artifacts, rendering its predictions plausible.

We proposed a straightforward, quantitative Explainability Accuracy (%) metric, defined as the ratio of samples for which Grad-CAM activations overlapped with dermatologist-annotated lesion masks by ≥ 70% (IoU ≥ 0.7). With a high inter-rater reliability ( κ=0.87), the model explainability of EnsembleSkinNet averaged 93.6% by class. The model’s visual reasoning in these findings matches that expected by an expert which supports the use of EnsembleSkinNet as a human-complementing, decision-support tool.

### 5.4 Bias, fairness, and dataset limitations

Although EnsembleSkinNet outperforms others both quantitatively and qualitatively, there are limitations which remain. While the HAM10000 dataset is an important step in this direction, it mainly consists of images from individuals with fair skin, and hence, remains unrepresentative from the perspectives of skin color and ethnicities. Such demographic biases might undermine generalization to populations that are not sufficiently represented in the training sets.

To counter this, future research will consist of:

- Include datasets from a wider variety of demographic and geographic contexts.
- Use bias-aware learning methods to equalize performance among subpopulations.
- Incorporate clinical metadata (e.g. patient age, lesion site and medical history) for a more informative diagnostic context.

Despite adding diversity to the dataset, augmentation cannot be a replacement for diverse, real world data. Cross-dataset validation (HAM10000 → ISIC 2020) is an initial step to assess these effects and shows the adaptability of the framework to heterogeneous data sources.

The use of weights pre-trained on ImageNet was effective as a transfer learning initialization, but it may not be reflective of the dermatological feature distribution. Domain-adapted pretraining on large-scale dermatology datasets (ISIC and Derm7pt) followed by specific fine-tuning strategies will be used in future work to improve feature specialization. And clinical metadata (e.g., lesion location, age, sex, and patient history) could additionally be incorporated into the ensemble model to bolster and interpret diagnostic performance—estimating accuracy with clinical metadata would allow the physician to correspond clinical risk characterizations directly to visual features.

### 5.5 Deployment considerations and model compression

Despite state of the art diagnostic performance among other ensembles like EnsembleSkinNet, its inherent computational complexity would hinder direct deployment on clinical-grade hardware. We then quantified the specific training and inference requirements and investigated model compression via knowledge distilling to a small student model.

#### 5.5.1 Computational complexity analysis

Average total number of parameters, floating-point operations (FLOPs) and inference latency across all evaluated models on 224×224 dermoscopic inputs were presented in **Table 11**. To calculate all metrics, an Nvidia RTX 3090 and Intel Core i9 was used.

**Table 11:** Computational complexity analysis.

Model	Parameters (M)	FLOPs (G)	Train time/Epoch (s)	Inference latency/Image (ms)
M-VGG16	17.8	3.9	52.1	64.3
ResNet50	23.5	4.1	58.6	62.5
Inception V3	24.0	4.3	61.7	65.2
DenseNet201	20.0	4.0	59.8	68.4
EnsembleSkinNet (4-model)	186.2	4.1 per image (≈ 16.4 total)	182.4	64.8
Distilled Student (MobileNet-V3-Small)	11.9	0.95	22.8	15.6

While EnsembleSkinNet combines multiple backbones, the shared feature extraction and softmax-weighted fusion maintain high throughput, with an inference latency of ≈ 65 ms/image — sufficient for offline or asynchronous clinical workflows.

To allow for real-time or mobile-edge deployment, knowledge distillation was used to develop a lightweight MobileNet-V3 student.

#### 5.5.2 Knowledge distillation framework

We performed teacher–student distillation, where the entire EnsembleSkinNet (teacher) gives soft labels and intermediary activations to the compact student.

Here, the distillation loss is as follows:


$$L_{KD}=(1-\alpha )L_{CE}(y,p_{s})+\alpha T^{2}KL(p_{t}^{T}\;\|\;p_{s}^{T})$$

where $p_{t}^{T}$ and $p_{s}^{T}$ denote teacher and student predictions softened by temperature T=3, and α=0.7 balances hard and soft supervision.

After distillation, the MobileNet-V3 student achieved:

#### 5.5.3 Clinical deployment feasibility

The distilled student model achieved 4× lower latency, 15× smaller parameter count, and ≈ 97% of the teacher’s accuracy, enabling deployment on edge GPUs, embedded AI systems (e.g., NVIDIA Jetson, Coral TPU), and hospital workstations as shown in **Table 12**.

**Table 12:** Teacher–Student distillation comparison.

Metric	Teacher (EnsembleSkinNet)	Student (MobileNet-V3)	Relative change
Accuracy (%)	98.32 ± 0.41	96.97 ± 0.44	–1.35
F1-score (%)	98.15 ± 0.37	96.88 ± 0.40	–1.27
AUC	0.983	0.974	–0.009
Inference (ms/image)	64.8	15.6	4.1 × faster

This confirms that EnsembleSkinNet can be translated into resource-constrained environments through compression, without sacrificing diagnostic reliability.

To further enhance scalability, future work will integrate:

- Model quantization (INT8 and FP16 inference).
- Edge AI optimization using TensorRT and ONNX Runtime.
- Federated deployment pipelines for multi-center learning.

### 5.6 Summary and future outlook

The proposed EnsembleSkinNet establishes a robust and interpretable foundation for AI-assisted dermatological analysis. Its primary achievements include:

- High diagnostic accuracy and robustness—98.32 ± 0.41% on HAM10000 and 96.84 ± 0.42% on ISIC 2020.
- Balanced per-class performance achieved through targeted augmentation (**Table 2**, **Figure 3**)
- Explainable predictions validated by Grad-CAM visualizations and quantitative metrics.
- Demonstrated cross-domain generalization that supports clinical translation.

In future work, EnsembleSkinNet will be part of a multimodal diagnostic framework incorporating dermoscopy images, histopathology, and clinical metadata through the application of transformer-based attention mechanisms. We believe that this, in combination with model compression and quantization, will allow for real-time inference on edge and mobile devices, which are essential for adoption in teledermatology and point-of-care screening. EnsembleSkinNet will be incorporated into a multi-modal diagnostic framework as part of future work to enable more contextual predictions by integrating dermoscopic images along with clinical, demographic, and if available, histopathological data. The ensemble strategy will also be expanded with attention-based or transformer-based fusion mechanisms to more dynamically model inter-feature relationships instead of using a static averaging mechanism. For enhanced generalization and scalability, we will extend the framework to federated learning as well as cross-dataset testing at multi-institutions for collaborative but privacy-preserving learning. In addition, model compression methods including pruning, quantization, and knowledge distillation would be utilized to optimize inference speed and deployment on clinical and edge devices. Such advancements can drive EnsembleSkinNet closer to clinical implementations, while providing diagnostic efficiency and interpretability and useful on all healthcare environments.

### 5.7 Conclusion of discussion section

EnsembleSkinNet highlights the potential of many-objective ensemble learning as a practical framework for jointly optimizing diagnosis performance and interpretability to produce skin-lesion analysis tools that may benefit both machine and human. This external validation, bias assessment, and quantitative explainability incorporate feedback from the reviewer, adding methodological rigor, transparency, and ultimately, feasibility for clinical dermatology to the framework.

## 6 Conclusion and future work

### 6.1 Limitations

Despite the high diagnostic performance of the proposed EnsembleSkinNet framework, the limitations of this study need to be addressed.

The present evaluation was not generalizable beyond HAM10000, as it was conducted within a specific patient population and imaging device setting.

- Section 4.3 (Additional validation on the ISIC 2020 dataset) helps alleviate this issue partially, but wider cross-institutional and multi-device testing is an important avenue of future work. As with other research protocols, clinical use of TTT will require consideration of several important issues.
- Including variability across devices, illumination uniformity, and the need for regulatory approvals to enable deployment into dermatologic workflows.
- The explainability evaluation is, although Grad-CAM visualizations were shown as compelling supplementary evidence, however lacks standardized quantitative benchmarks across the literature, motivated by a qualitative focus (v). Moving forward, we will investigate incorporating human–AI evaluation protocols and formal interpretability assessment standards.
- Even with extensive augmentation, residual risk of overfitting (class imbalance, homogeneity of the dataset). Further iterations of the framework will integrate more regularization techniques and cross-domain adaptation to boost robustness even more.
- Lastly, this study has not compared to classical ML models like SVM or Random Forest. Few studies will apply these baselines using CNN-extracted feature vectors to enhance comparative validity in future research.

Improving these limitations will increase the clinical robustness, generalizability and interpretability of the framework in a range of out-of-the-lab environments.

### 6.2 Conclusion

To do so, this work presents EnsembleSkinNet, a novel framework to detect skin cancer that uses an ensemble learning strategy over several pre-trained models. The model also obtains a higher accuracy, precision, recall, and F1-score than current individual and ensemble methods. EnsembleSkinNet overcomes most of the domain challenges from the literature by utilizing transfer learning, applying fine-tuning and modeling averaging to handle complex manifestation variations as well as being more generalizable over different datasets. Our experimental results validate the model potential, which will be a good step for improving skin cancer diagnostics, through inherently more robust and accurate automated skin lesion classification with capability for better detection of skin lesions. Comparatively, the study has some limitations including the usage of different computational resources in providing the multiple models, the functioning on extremely small or imbalanced datasets and also no testing on the actual clinical data. Their capabilities are stronger — but also limited — and we still need to explore the breadth of their potential and the best methods of utilization. More optimizations can be performed on the model to make it efficient like minimizing the computation cost while maintaining the performance. Extending generalizability assessment of the framework on more diverse clinical datasets would also be valuable to validate the framework. Nonetheless, the proposed model as such lacks robustness and may be improved by including multi-modal data which researchers incorporated in other studies specifically patient clinical history or ceroscopy features.

## Data Availability

Publicly available datasets were analyzed in this study. This data can be found here: https://www.kaggle.com/datasets/kmader/skin-cancer-mnist-ham10000.
